# Biogenic gold nanoparticles conjugated with rhizobacteria enhance tomato growth and suppress pathogen infection

**DOI:** 10.3389/fmicb.2026.1758150

**Published:** 2026-05-05

**Authors:** Narjes Haje Dashti, Nedaa Ali, Anandavalli Inbamani Raju

**Affiliations:** 1 Department of Biological Sciences, Faculty of Science, Kuwait University, Sabah Al-Salem University City, Al-Shaddadiya, Kuwait; 2 College of Science, Kuwait University, Khaldiya, Kuwait

**Keywords:** disease suppression, *Erwinia persicina*, gold nanoparticles (AuNPs), nanobio conjugation, plant growth-promoting rhizobacteria, rhizosphere colonization, *Stenotrophomonas rhizophila*

## Abstract

*Erwinia persicina* (Ep) is recognized as an emerging pathogen of considerable threat to tomato yields. Although *Stenotrophomonas rhizophila* (Sr) has long been recognized as a plant growth-promoting rhizobacterium (PGPR), the use of this PGPR has sometimes been constrained by the rhizosphere stress factor in the biocontrol of the pathogen. This study investigated the potential of biogenic gold nanoparticles (AuNPs) to enhance the performance of Sr. through bio-conjugation. Biogenic AuNPs were synthesized and conjugated with *S. rhizophila* (Sr-AuNPs). The physical and biochemical stability of the conjugate was verified via TEM, DLS, and FTIR. Greenhouse trials were conducted across three independent growing seasons to evaluate the impact of the Sr-AuNP conjugate (T3) on tomato growth, yield, and resistance against *E. persicina* infection (T4) compared to healthy controls (T1) and non-conjugated bacteria (T2). Bio-conjugation facilitated robust root surface colonization, with FESEM-EDS confirming AuNP persistence (7.1–10.2 wt.%) on the root epidermis. The T3 treatment consistently demonstrated superior disease suppression, maintaining the lowest Disease Severity Index (DSI) values (0.65–0.80) throughout the study. Agronomically, T3 was the only treatment that sustained the highest statistical order (Tukey group ‘a’) in terms of fruit weight throughout the three seasons, reaching 204.62 g in Season 3, which was a remarkable threefold increase from the infected control T4 (72.52 g). These results indicate that AuNPs act as a supporting scaffold that promotes the longevity and efficacy of PGPR. Interestingly, rather than acting solely as a plant growth promoter, the bio-conjugate functioned chiefly as a potent disease suppressor and plant growth stabilizer under high pathogen pressure. This biologically derived method presents itself as a sustainable solution to the threat presented by emergent phytopathogens in contemporary agriculture.

## 1 Introduction

Modern-day agriculture is facing a twofold challenge: maintaining crops productivity and simultaneously cutting down the use of chemical fertilizers and pesticides. These chemicals harm the environment and people’s health. Plant diseases caused by bacterial pathogens are still one of the major reasons for reduced agricultural production. This is especially true in areas with intensive farming, high pathogen pressure, and limited chemical control options. Tomato (*Solanum lycopersicum* L.) is one of the most widely cultivated vegetables and one of the most valuable crops globally. It has an economic value that goes beyond its nutritional importance as it provides a significant source of vitamins, antioxidants, and bioactive compounds for human ingestion (Ali et al., 2020). However, tomatoes are highly susceptible to a range of bacterial, fungal, and viral pathogens that significantly impact both yield and fruit quality. This vulnerability poses a major challenge for successful cultivation and production. The bacterial pathogen *Erwinia persicina* has emerged as one of the main causal agents of soft rot and vascular infection in tomato. These infections lead to significant economic losses for several production systems (González et al., 2007; Wasendorf et al., 2022; Millett et al., 2025). The gradual decrease in the use of chemical bactericides along with the application of stricter regulatory frameworks has created an urgent need for effective, sustainable, and biologically-based disease management strategies. This emphasized the importance of developing and adopting effective, sustainable, and biologically based approaches.

Plant growth-promoting rhizobacteria (PGPR) are an effective approach for controlling pests and diseases in agriculture without the use of chemicals (Khanna et al., 2019; Santoyo et al., 2021; Wang et al., 2021; Yang et al., 2024). Therefore, they have garnered great interest in recent years. These friendly bacteria live in soil alongside the plant roots and enhance the plants’ resistance both externally and internally. They achieve this through various mechanisms such as improved nutrient absorption, hormone secretion, modulation of plant stress response, and suppression of pathogens (Nelson, 2004; Vacheron et al., 2013; Compant et al., 2019; Chandran et al., 2021; Panichikkal and Krishnankutty, 2022; de Andra et al., 2023). These microorganisms facilitate reduced fertilizers use, leading to the production of better-quality crops and less nature pollution. However, the achieved success observed in the field and greenhouse experiments have often been limited and inconsistent. The main reason for this inconsistency is the heavy competition with native soil microorganisms, changing environmental conditions, and the lack of stable colonization in the roots. Together, these factors make it difficult for the introduced strains to survive and work effectively (Backer et al., 2018).

Among the diverse PGPR taxa, *Stenotrophomonas rhizophila* has emerged as a promising rhizobacterium with strong plant growth-promoting and stress-mitigating properties. This Gram-negative, fluorescent bacterium is well adapted to the rhizosphere. It has been reported to promote plant growth by improving nutrient availability, producing bioactive metabolites, and suppressing pathogenic microorganisms (Wolf et al., 2002; Schmidt et al., 2012; Dashti et al., 2021; Jiao et al., 2021). In addition to its role in plant growth promotion, *S. rhizophila* participates in nutrient cycling. It also exhibits bioremediation capabilities, including the degradation of organic pollutants such as phenols, polycyclic aromatic hydrocarbons, and aromatic hydrocarbons (Radwan et al., 2005; Radwan et al., 2007; Dashti et al., 2009).

Despite these advantages, the practical application of *S. rhizophila* in agriculture is constrained by challenges related to consistent root recolonization and survival under competitive and stress-prone soil environments. The recent breakthroughs in nanotechnology have led to the development of new methods that would improve the performance of inoculants in agriculture (Uz et al., 2016; Shang et al., 2019; Kenawy et al., 2019; Duhan et al., 2021; Khalid et al., 2022; Zafar et al., 2024). The unique physicochemical properties of nanomaterials, especially metal nanoparticles, include their size range of 1–100 nm, high surface-to-volume ratio, tunable surface chemistry, and size-dependent reactivity (Joudeh and Linke, 2022; Tandiana et al., 2022). These physicochemical properties of the metal nanoparticles make them very efficient in interacting with the biological systems like the plant roots and the microbial cells (Siddiqi and Husen, 2017; Lead et al., 2018; Timmusk et al., 2018). Gold nanoparticles (AuNPs) are among the most prominent metal nanoparticles owing to their remarkable characteristics, including chemical stabilization, non-toxicity to living organisms, and their compatibility with biological systems (Khan et al., 2022). Additionally, they can be readily surface functionalized, enhancing their versatility in various applications (de Moraes et al., 2021; Saputri et al., 2025). Their attributes have enabled numerous applications in different fields, Including biomedicine, environmental cleaning, and development of biosensors. They are also used in systems for the controlled delivery of antimicrobial agents (Dykman and Khlebtsov, 2012; Gul et al., 2025).

Green synthesis techniques utilizing biological materials for the reduction and stabilization of nanoparticles have attracted attention of researchers. This approach offers a means to address the environmental and safety concerns linked to conventional methods of nanoparticle synthesis (Singh et al., 2016; Ramakrishna et al., 2016; Dahoumane et al., 2017; Khan et al., 2019; Abbasifar et al., 2020; Mukherjee et al., 2021).

Among various alternatives, the production of gold nanoparticles (AuNPs) using marine macroalgae has been particularly highlighted. This method is noted for its low environmental impact and the potential to scale the process and thus meet global demand (Dahoumane et al., 2017; Khan et al., 2019). Marine macroalgae contain a wide variety of natural compounds including polysaccharides, phenolic compounds, terpenoids, proteins. These compounds not only reduce metal ions to form nanoparticles but also stabilize and provide biological functionality to the resulting AuNPs (Anuradha et al., 2015; Ramadhani and Said, 2024). Among these, green seaweed *Caulerpa sertularioides* has emerged as a source of promising compound with a rich secondary metabolite pool that can reduce and stabilize metal ions (Rajeshkumar et al., 2013; Sri Sivakumar et al., 2025). However, its application in the synthesis of bio-conjugated nanoparticles for disease suppression remains under-explored. When compared to chemically synthesized AuNPs, AuNPs produced using biological methods display increased compatibility with living organisms and therefore, create less environmental risk to popery producers (Panichikkal et al., 2019; Lee et al., 2020). Bioregenerative nanoparticles in plant systems have been shown to influence physiology and biochemical processes, such as redox regulation, chlorophyll production, and nutrient acquisition. These effects enhance plant resilience against abiotic factors such as salinity stress and drought (Rasheed et al., 2022; Amir et al., 2024; Javaid et al., 2024; Ruffatto et al., 2025; Tiwari et al., 2025).

Bioregenerative evidence suggests that nanoparticles can influence plant microbial interactions by serving as a bioscaffold for microbial attachment to root surfaces. They also promote microbial biofilm formation and enhance microbial survivability within the rhizosphere (Milewska-Hendel et al., 2021; Bhatia et al., 2021). However there has been very scant research regarding the potential of bio-AuNPs to act as a performance-enhancing tool for PGPR under biological stress such as bacterial infection. Accordingly, the main goal of the present study was to investigate whether biogenic gold nanoparticles, synthesized using the marine macroalga *Caulerpa sertularioides* can enhance the growth-promotion and biocontrol potential of *Stenotrophomonas rhizophila* against the pathogenic bacterium *Erwinia persicina* in tomato plants. The biosynthesized AuNPs were characterized from a physicochemical viewpoint, and their conjugation with *S. rhizophila* was assessed. Greenhouse experiments were carried out for successive growing seasons to evaluate the effects of this nano–biological system on tomato plants growth. The study also assessed disease severity, pathogen load, root colonization, and gold accumulation was appraised. By combining green nanotechnology with microbial biocontrol, the present work confers novel insights into a sustainable and targeted approach for bacterial disease management in tomato.

## 2 Materials and methods

### 2.1 Biosynthesis and characterization of gold nanoparticles (AuNPs)

Gold nanoparticles (AuNPs) were synthesized using a green method wherein the native marine macroalgae, *Caulerpa sertularioides*, acted as the reducing and stabilizing agent. The reaction mixture consisted of aqueous *C. sertularioides* extract (1.5% w/v) and gold (III) chloride (AuCl₃, 1.0 mM) prepared at a 1:1 (v/v) ratio and kept in the dark for 18–24 h at room temperature (25 ± 2 °C). The color changes from pale yellow to ruby red was an indication of the formation of AuNPs. The nanoparticles were collected by centrifugation at a speed of 15,000 rpm for 10 min at 20 °C. Then, they were washed three times with distilled water, dried in an oven for 24–48 h and kept at 4 °C until further usage. The produced AuNPs were analyzed through UV–Vis spectroscopy, X-ray diffraction (XRD), X-ray photoelectron spectroscopy (XPS), zeta potential analysis, thermogravimetric analysis (TGA), transmission electron microscopy (TEM), scanning electron microscopy (SEM), and Energy Dispersive X-ray spectroscopy (EDS) according to Montasser et al. (2017).

### 2.2 PGPR strain source and inoculum preparation

The study utilized a plant growth–promoting rhizobacterial (PGPR) strain, which was previously derived from fava bean (*Vicia faba* L.) rhizosphere soil through the standard methods of serial dilution and plating. The isolate was recognized as *Stenotrophomonas rhizophila* strain GSB-381 through 16S rRNA gene sequencing, and the sequence was submitted to the NCBI GenBank database (Accession No. MK161197). The phylogenetic study (NCBI Neighbor joining tree) showed that the MK161197.1 isolate strongly matches *Stenotrophomonas rhizophila*. The PGP trait of *S. rhizophila* GSB-381 was estimated by the production of Indole-3-Acetic Acid (IAA) Production using the Salkowski colorimetric assay, following the specific reagent-to-supernatant ratio described by Gang et al. (2019) (Supplementary Figure S1 and Supplementary Table S1A).

For the preparation of the inoculum, *S. rhizophila* GSB-381 was grown in nutrient broth and incubated at 31 °C with constant shaking (125 rpm). The cultures were taken at the logarithmic growth phase and were adjusted spectrophotometrically to OD₆₀₀ = 0.1 with sterile distilled water. Taking into consideration, the species-specific variations in the relationship of optical density to cell number, a calibration curve specific to the strain was made by serial dilution and plate counting. Following this calibration, OD₆₀₀ = 0.1 was found to be about 10^8^ CFU/mL for *S. rhizophila* under the experimental conditions applied (Supplementary Table S1B).

### 2.3 Growth response of *Stenotrophomonas rhizophila* to AuNPs

The tolerance of *S. rhizophila* to various concentrations (100–1,000 μg/mL) of AuNPs was determined via a Bioscreen C analyzer (Oy Growth Curves Ab Ltd., Finland). Bacterial cultures (OD₆₀₀ = 0.1) were mixed at a 1:1 (v/v) ratio with suspensions of AuNPs in 100-well microplates and incubated at 37 °C for 24 h. The wells with bacteria alone served as a control. The optical density at 600 nm was measured every hour, and growth curves were plotted as the means of three independent replicates.

### 2.4 Preparation of AuNPs–conjugated (Sr–AuNPs)

*Stenotrophomonas rhizophila* was conjugated with AuNPs naturally in the absence of any chemical additives. Based on growth kinetic studies, AuNPs at concentrations of 100 μg /mL were chosen for conjugation with *S. rhizophila*, as this concentration did not inhibit bacterial growth and maintained cell viability for subsequent plant studies. To this end, a 1:1 v/v bacterial suspension (10^8^ CFU /mL) and AuNPs (100 μg/mL) were mixed and incubated at 31 °C for 30 min under gentle shaking (100 rpm). Polysaccharides and proteins from *C. sertularioides* serve as natural capping and adhesive agents, respectively, promoting stable binding of nanoparticles on bacterial surfaces.

### 2.5 Characterization of Sr–AuNPs conjugates

The conjugation efficiency and surface modifications of the Sr–AuNPs were analyzed via Fourier-transform infrared spectroscopy (FTIR), dynamic light scattering (DLS), field emission scanning electron microscopy–energy dispersive spectroscopy (FESEM-EDS), and transmission electron microscopy (TEM) techniques. FTIR spectroscopy was carried out using a Jasco FT/IR-6300 spectrophotometer with oven-dried samples (1–5 mg, 45 °C). Spectra were recorded between 4,000–400 cm^−1^ with a 4 cm^−1^ resolution. Bands were assigned to O-H/N-H, C-H, Amide I, Amide II, COO^−^, and polysaccharide C-O. Shifts in bands and changes in intensity were used to determine functional group involvement in conjugation. Hydrodynamic diameter and poly dispersity index (PDI) were determined using DLS, while zeta potential was determined using a Zetasizer Nano ZS (Malvern Instruments, United Kingdom). Each measurement was done in triplicate, with mean values derived from 10 readings per replicate. FESEM-EDS was used to study surface morphology using a JEOL JSM-7001F, while liquid samples (2–5 μL) were air-dried, sputter-coated, and viewed to confirm gold deposition. Surface morphology was viewed using FESEM, while elemental composition was determined using EDS. TEM was carried out using a JEOL JEM-1400 Flash, where liquid samples (2–5 μL) were dropped onto carbon-coated grids, air-dried, and viewed to determine nanoparticle size, morphology, and adhesion to bacterial cells.

### 2.6 SEM and TEM analysis

Sample preparation for SEM and TEM followed Pajerski et al. (2019) with minor modifications. The bacterial cells of *S. rhizophila* (Sr), both in their unmodified state (T2 inoculum-Section 2.2) and following the bioconjugation process with gold nanoparticles (Sr–AuNPs) (T3 inoculum-Section 2.4) were fixed in 3% glutaraldehyde for 24 h, washed with phosphate-buffered saline (PBS), dehydrated in a graded ethanol series (50–100%), and coated with platinum (~15 nm) via a Quorum Q150T S sputter coater. Imaging was performed via field emission SEM (JEOL JSM-7001F, Japan).

For TEM, the cells were fixed in 3% glutaraldehyde, fixed in 1% osmium tetroxide, dehydrated through an ethanol gradient, and mounted on carbon-coated copper grids. Observations were made via transmission electron microscopy (TEM) (JEOL 1200, Japan) at 120 kV.

### 2.7 Phytopathogen source and inoculum preparation

The phytopathogen *Erwinia persicina* (strain USTRW7), which was originally isolated from a lignified natural product, was employed in this research work. The taxonomic identity was certified by the sequencing of the 16S rRNA gene (NCBI GenBank Accession No. KU923347). For inoculum preparation, the stock cultures that were kept at −70 °C were first streaked onto nutrient agar and incubated at 31 °C for 24 h. After this, one colony was taken and transferred into 100 mL of nutrient broth, and the microorganisms grew at 31 °C with constant shaking (100 rpm). The cells were collected during the logarithmic growth phase. To determine cell density accurately, a strain-specific calibration curve was generated using serial dilutions and viable plate counting. Based on this calibration, the culture was adjusted with sterile deionized water to an OD_600_ = 0.1, corresponding to a final concentration of approximately 10^8^ CFU/mL (Supplementary Table S1B).

### 2.8 Greenhouse conditions

The experiments were conducted in the Horticulture and Greenhouse Unit, Kuwait University, under controlled environmental conditions (25–28 °C; 25–30% relative humidity; continuous light). Cooling systems were used to maintain temperature stability during peak summer periods.

### 2.9 Plant material and growth conditions

Tomato (*Solanum lycopersicum* L.) cv. GVS 51306 Super Marmande was used as the model plant. Seeds were surface sterilized with 2% sodium hypochlorite containing Tween 20 (Radwan et al., 2005), rinsed thoroughly with sterile distilled water and sown in a sterilized mixture of peat moss and perlite (3:1 v/v). Seedlings were acclimatized in the greenhouse for 48 h and irrigated with Hoagland’s nutrient solution (Hoagland and Arnon, 1950).

### 2.10 Experimental design

A randomized complete block design (RCBD) was used, with four treatments (20 plants each) replicated across three growing seasons:

- T1 (Control): Untreated plants.
- T2 (Sr + Ep): *S. rhizophila* + *E. persicina.*
- T3 (Sr–AuNPs + Ep): AuNP–conjugated *S. rhizophila* + *E. persicina.*
- T4 (Ep): *E. persicina* only.

Bacterial inoculations (10 mL/plant, 10^8^ CFU/mL) were applied at the dicotyledonary stage. Pathogen challenge with *E. persicina* (10^8^ CFU/mL) was performed 2 weeks later. The plants were maintained under 16-h light/8-h dark cycles and irrigated with sterile Hoagland’s nutrient solution.

### 2.11 Plant growth and disease assessment

At 120–125 days after transplantation, the plant height, fresh weight, dry weight, and fruit weight were recorded. Fresh biomass was measured immediately, while dry biomass was determined after shade-drying at 25–28 °C for 8–10 days.

Disease severity caused by *E. persicina* was scored 7 days post-inoculation on a 0–5 scale (0 = no symptoms, 5 = plant death). The disease severity index (DSI) was calculated for each treatment.

### 2.12 Rhizospheric bacterial population assessment

Rhizosphere soil was collected from five randomly selected plants within each treatment group: T1 (Control), T2 (Sr + Ep), T3 (Sr–AuNPs + Ep), and T4 (Ep only) at the end of each growing season. Plants were gently pulled up, and soil well attached to the roots was gently shaken out into sterile vials to collect rhizosphere soil. Root samples were rinsed with sterile phosphate-buffered saline (PBS) to dislodge loosely adherent microorganisms. The PBS-washed root samples were subsequently homogenized with sterile PBS, and suspensions were then challenged using serial dilutions (10^−1^ to 10^−6^), as described by Glick (2012). An aliquot of 100 μL from all dilutions was plated twice on nutrient agar plates. Plates were incubated at 31 °C for 24–48 h. Colony-forming units (CFUs) were counted and expressed on a fresh weight basis as CFUs/g rhizosphere soil (Stockdale and Brookes, 2006).

### 2.13 Detection of gold in root clusters via ICP–MS

The presence of AuNPs in the root cluster was detected using ICP-MS. The root cluster samples were removed from plants, air-dried in the dark at room temperature for 8–10 days, and then oven-dried to complete dryness. The homogenized samples were ground into fine powder with a sterile mortar and pestle and analyzed for elemental composition by microwave-assisted digestion (Titan MPS, PerkinElmer) following Bettinelli et al. (2005). Approximately 0.5 g of root tissue that had been ground was weighed into a digestion vessel to which 9 mL of concentrated HNO₃, 1 mL of HCl, and 1 mL of H₂O₂ were added. Two consecutive heating 303 steps of microwave digestions were performed: first at 175 °C for 10 min, and second at 220 °C for 10 min. The digested solution was filtered through Whatman No. 42 filter paper 305 and diluted to a final volume of 50 mL with grade 1 deionized water (18.2 MΩ). Elemental 306 gold concentration was measured with an ICP-MS instrument (NexION 2000P, PerkinElmer) that had been calibrated against a series of certified gold standards (0, 0.1, 0.5, 1.0, 5.0, and 10.0 ppb) and reagent blanks. Instrument conditions were plasma gas flow 18 L/min, auxiliary 309 gas flow 1.2 L/min, nebulizer gas flow 0.92 L/min, and RF power 1,600 W. A PFA-ST3 310 nebulizer, quartz cyclonic spray chamber, high-efficiency quartz torch with quartz injector 1 mm, and nickel cones were used during analysis. The error margin of the method was 10%.

### 2.14 FESEM–EDS analysis of root colonization

The root samples were prepared for FESEM imaging following the method described by Gamez et al. (2019), with an additional fixation step. Five to six tomato root samples were randomly collected after 5 days in the proliferation tray, prior to the addition of *E. persicina* (Ep), from plants treated with *S. rhizophila* (Sr) or Sr. bioconjugated with gold nanoparticles (Sr–AuNPs). The roots were carefully washed in water to remove excess substrate, ensuring that the attached microorganisms were not disturbed. The roots, with Sr. colonization, were then immersed in a fixative solution (2.5% glutaraldehyde in phosphate buffer) and refrigerated for at least 4 h. After fixation, the roots were dried at 45 °C for 3 days and then cooled to 4 °C for further processing. To prepare the samples for FESEM imaging, the roots were first dehydrated with 20% ethanol and subsequently rinsed with acetone. The dried root samples were then mounted on aluminum stubs using carbon adhesive tapes and coated with platinum (Pt) using a Quorum Technologies Sputter Coater Q150. The Pt-coated samples were analyzed using the Field Emission Scanning Electron Microscope JEOL (JSM-7001F, Japan) with Energy Dispersive X-ray Spectrometer (EDS) (INCAx-act, Oxford Instruments, United Kingdom) used for imaging, and element analysis (% wt.) of the samples, respectively, with a typical vacuum of 10–5 Pa. FESEM images of T2 (Sr) and treatment 3 (Sr–AuNPs+Ep) root samples were captured at various magnifications to observe bacterial colonization and the root surface morphology.

### 2.15 Quantification of *Erwinia persicina* by real-time qPCR

Genomic DNA was isolated from tomato leaves infected with *E. persicina* using PrepMan™ Ultra Sample Preparation Reagent following the manufacturer’s protocol, where approximately 100 mg of infected leaf tissue was homogenized, heated at 100 °C for 10 min, and centrifuged to collect the supernatant for immediate qPCR or storage at −20 °C. Specific primers targeting the 16S rRNA gene of *E. persicina* (strain USTRW7; GenBank accession no. KU923347.1) were designed via Primer- BLAST, with Primer set 3 selected for optimal melting temperature and amplification efficiency; sequences are provided in Supplementary Table S2.

Quantitative real-time PCR was performed using a ViiA™ 7 Real-Time PCR System with SYBR™ Green detection chemistry in 20 μL reactions containing 10 μL of Master Mix, 0.5 μM of each primer, and 1 μL of DNA template. The thermocycling conditions consisted of 95 °C for 10 min followed by 40 cycles of 95 °C for 15 s and 60 °C for 1 min, concluded by a melting curve analysis to ensure specificity. Absolute quantification was achieved using a standard curve generated from genomic DNA of pure *E. persicina* cultures, serially diluted threefold from an initial 50 ng μL^−1^, with amplification efficiency and R^2^values calculated automatically to determine the bacterial DNA load in the infected samples.

### 2.16 Statistical analysis

All experiments were conducted in triplicate across three independent growing seasons to ensure reproducibility. Data are expressed as means ± standard errors of the mean (SEM). Statistical significance among treatment groups was assessed by one-way analysis of variance (ANOVA) followed by Tukey’s honestly significant difference (HSD) *post hoc* test (*p <* 0.05). Pairwise comparisons were further evaluated using the studentized range (Q) statistic, with significance set at *p <* 0.01. All quantitative data, including growth parameters and fruit weight, were assigned a compact letter display (CLD), where treatments sharing the same letter are not significantly different. Analyses were performed using Microsoft Excel for Microsoft 365 (Version 2,510 Build 16.0.19328.20144; Microsoft Corporation) and the astatsa Online Statistical Calculator^1^. To assess the relationship between rhizospheric populations and *E. persicina* abundance, Spearman’s rank correlation coefficient (rs) was calculated using the mean values of each treatment (*n* = 4). *p*-values < 0.05 were considered statistically significant.

## 3 Results

### 3.1 Physicochemical and structural characterization of biosynthesized gold nanoparticles

**Figure 1:**
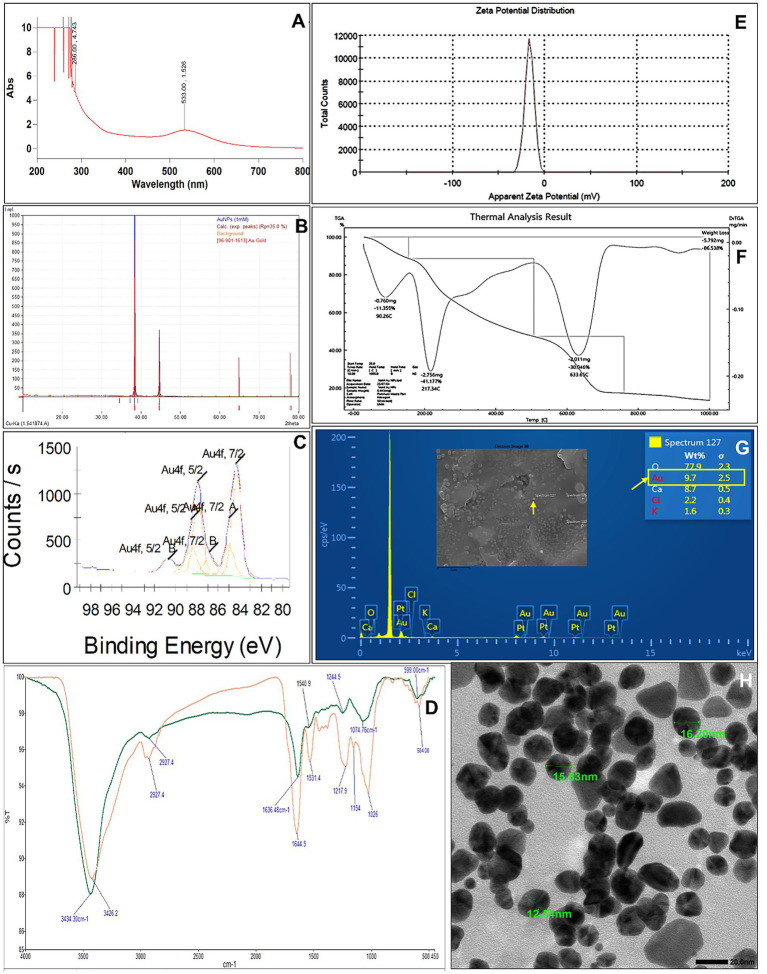
Characterization of the biosynthesized AuNPs. **(A)** UV–VIS spectrum. **(B)** XRD pattern. **(C)** XPS spectrum. **(D)** FTIR spectra of the algal extract (green) and synthesized AuNPs (orange). **(E)** Zeta potential distribution. **(F)** TGA profile. **(G)** FESEM-EDS mapping of the elemental composition of the AuNPs. **(H)** TEM image. Magnification-100,000×; scale bar = 20 nm.

UV–visible spectroscopy revealed a prominent localized surface plasmon resonance (LSPR) peak at 533 nm, confirming successful AuNP formation (Figure 1A). XRD analysis revealed characteristic diffraction peaks at 2θ values of 38.2°, 44.4°, 64.6°, and 77.0°, corresponding to the (111), (200), (220), and (311) planes of face-centered cubic (FCC) gold, respectively (Figure 1B). The XPS spectrum exhibited Au 4f₇/₂ and Au 4f₅/₂ peaks at ~84 eV and ~88 eV, respectively, verifying the metallic state of gold (Figure 1C). The FTIR spectra of the algal extract and AuNPs revealed bands at 3324–3388, 1,650, 1,410, 1,250, 1,150, 1,076, and 579 cm^−1^, confirming the role of algal biomolecules in nanoparticle reduction and stabilization (Figure 1D). The zeta potential of −16.4 mV indicated good colloidal stability (Figure 1E). Thermogravimetric analysis (TGA) revealed major weight losses at 90.26, 217.34, and 633.65 °C, corresponding to water loss, decomposition of organic components, and thermal degradation of residual biomolecules, respectively (Figure 1F). FESEM–EDS confirmed the elemental presence of Au (9.7 wt%) along with N, Ca, Cl, and K, suggesting organic capping by algal metabolites (Figure 1G). TEM images revealed uniformly distributed spherical-to-elliptical nanoparticles, 12.5–16.2 nm in size (Figure 1H). Collectively, these analyses confirmed the crystalline, stable, and biologically capped nature of the AuNPs synthesized using the native green alga *C. sertularioides*.

### 3.2 Growth response of *Stenotrophomonas rhizophila* to gold nanoparticles

**Figure 2:**
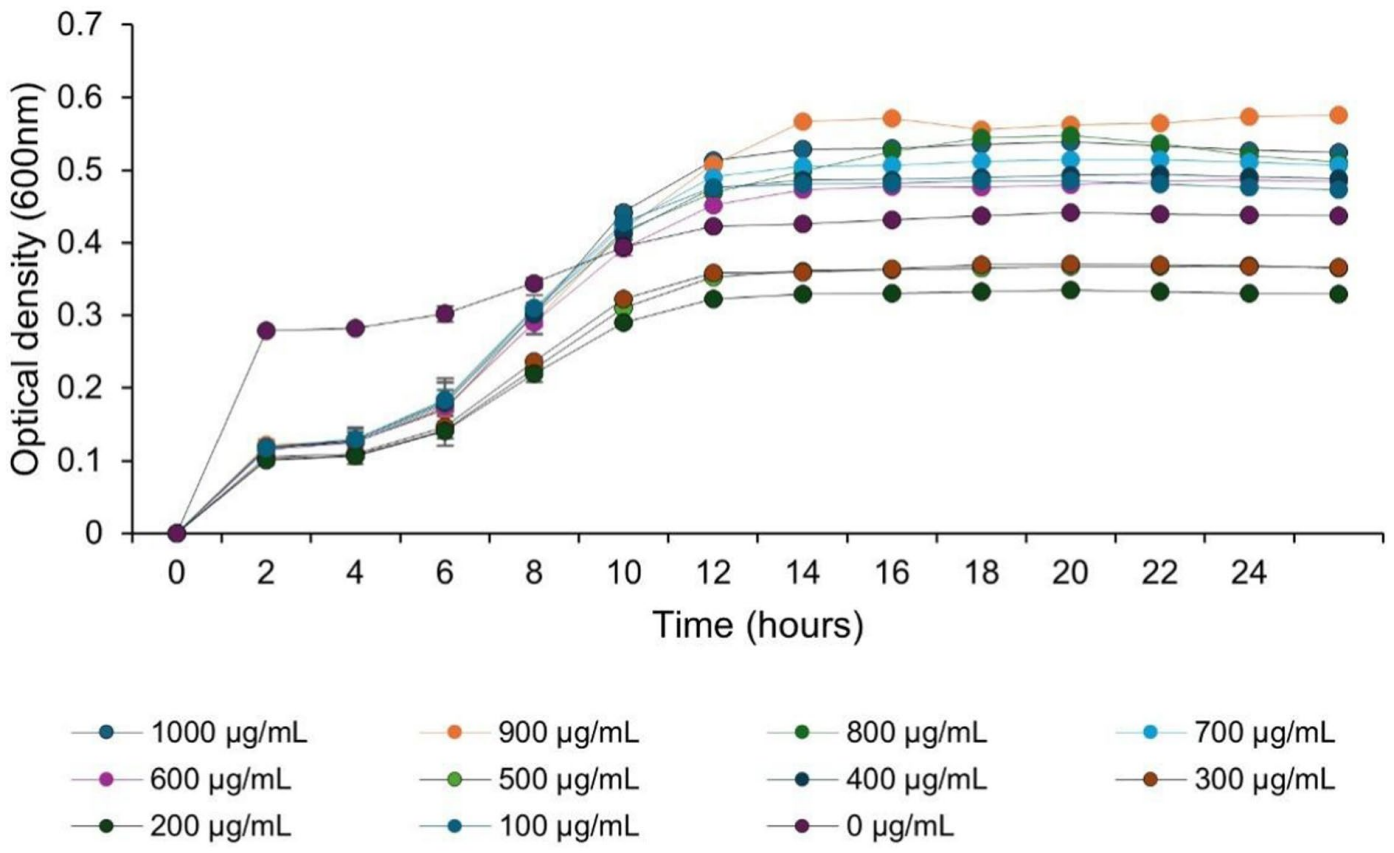
Growth response of *Stenotrophomonas rhizophila* to various concentrations of AuNPs. The OD_600_ values were recorded over 24 h at 30 °C in nutrient broth to evaluate the bacterial response to AuNPs (0–1,000 μM). The data represent the means ± SDs (*n* = 3).

The relationship between AuNPs concentrations and the growth kinetics of *S. rhizophila* is illustrated in Figure 2, using the OD₆₀₀ measurement to evaluate bacterial growth for a time frame of 24 h. OD₆₀₀ results from the experiments demonstrated that the growth of the bacteria was dependent on the amount of AuNPs that they were exposed to throughout the course of the experiment over the additional time. Bacterial cultures exposed to AuNPs at concentrations between 100 and 400 μg/mL had bacterial growth rates that were similar to, or slightly greater than, the growth of cultures that were not treated with AuNPs (i.e., controls) as evident by a final OD₆₀₀ value of around 0.48–0.55 in the stationary phase. On the other hand, exposure to AuNP concentrations greater than or equal to 500 μg/mL resulted in reduced bacterial growth (i.e., decreased bacterial OD₆₀₀ values and less time spent in the exponential phase of the growth curve). Bacterial growth was significantly inhibited by the highest amounts of AuNP tested (700–1,000 μg/mL), and even under this level of treatment measurable increases in OD₆₀₀ were observed regardless of whether the *S. rhizophila* were exposed to AuNPs at lower or higher concentrations, however cell densities were significantly decreased compared to controls at max concentration. Overall, these findings demonstrate that AuNPs can alter the growth kinetics of *S. rhizophila*.

### 3.3 Biophysical and biochemical characterization of the *Stenotrophomonas rhizophila*-AuNP conjugate

**Figure 3:**
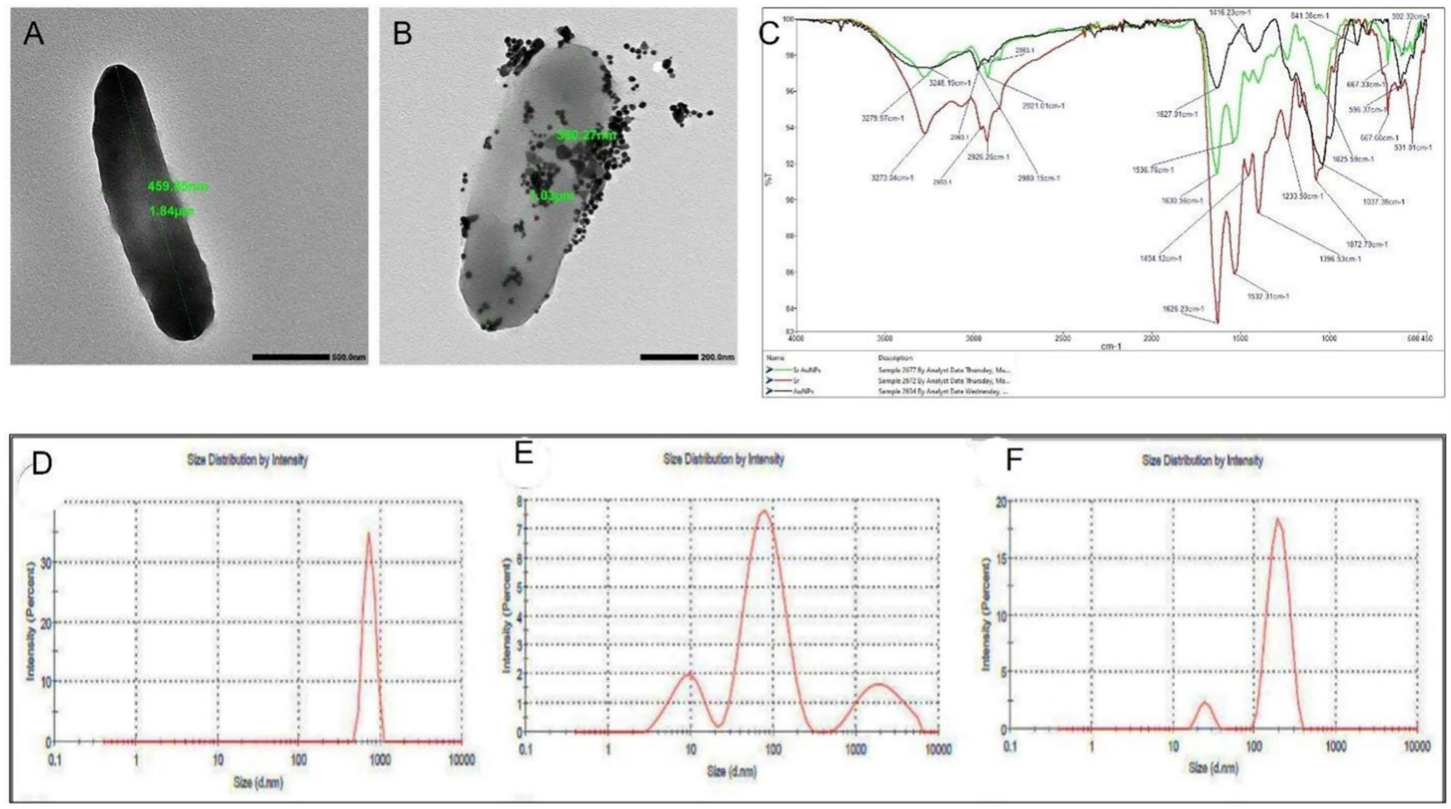
Morphological and biophysical characterization of the *Stenotrophomonas rhizophila*–AuNP conjugate. **(A,B)** TEM micrographs of unmodified *S. rhizophila* (*Sr*) and the *Sr*–AuNP conjugate (scale bars = 200 nm). **(C)** FTIR spectra of *Sr*, AuNPs, and the *Sr*–AuNP conjugate, showing new vibrational bands at 1396 cm^−1^ and 548 cm^−1^ associated with metal–ligand bonding. **(D–F)** DLS hydrodynamic diameter distributions for *Sr* cells, AuNPs, and *Sr*–AuNPs.

The TEM analysis showed that the untreated cells of *S. rhizophila* had smooth, rod shape measuring 1.8 to 2.1 μm in length (Figure 3A). After conjugating the cells with AuNPs, visible clusters of nanoparticles could be seen attached to its cell wall, with minimal effects on its morphology, thus emphasizing the efficiency of the conjugation and the compatibility of the synthesized AuNPs with the cells (Figure 3B).

Biochemical changes during the conjugation process were verified using FTIR spectroscopy (Figure 3C). The FTIR spectrum for the Sr-AuNP conjugate is represented by the red line and exhibited considerable differences in the spectrum compared to the control Sr. spectrum represented by the green line and chemically synthesized AuNPs represented by the black line. The broad absorption peak at 3273 cm is due to the stretching of the O-H bond in phenolic compounds and N-H bonds in primary amine groups. The sharp absorption peaks at 1626 and 1,532 cm for Amide I and Amide II bonds indicate the presence of carbonyl groups and N-H bonds in peptide linkages. This suggests that proteins and glycoproteins play an important role as the major stabilizing agents for the nanoparticles. The peak at 1037 cm indicates the involvement of C-O-C bonds in polysaccharide groups in the stabilization process.

Biophysical characterization with Dynamic Light Scattering (DLS) analysis further supported surface interactions (Figures 3D–F). The AuNPs alone demonstrated a hydrodynamic diameter of 48.84 nm (PdI = 0.748; *ζ* = −14.2 mV) (Figure 3D). The *S. rhizophila* cells without any modifications demonstrated a hydrodynamic diameter of 732.8 nm (PdI = 0.201; ζ = −15.7 mV) compared to a conjugated size change to 194.4 nm (PdI = 0.338; ζ = −16.4 mV) for Sr-AuNPs.

### 3.4 Greenhouse evaluation of plant growth parameters across seasons

**Figure 4:**
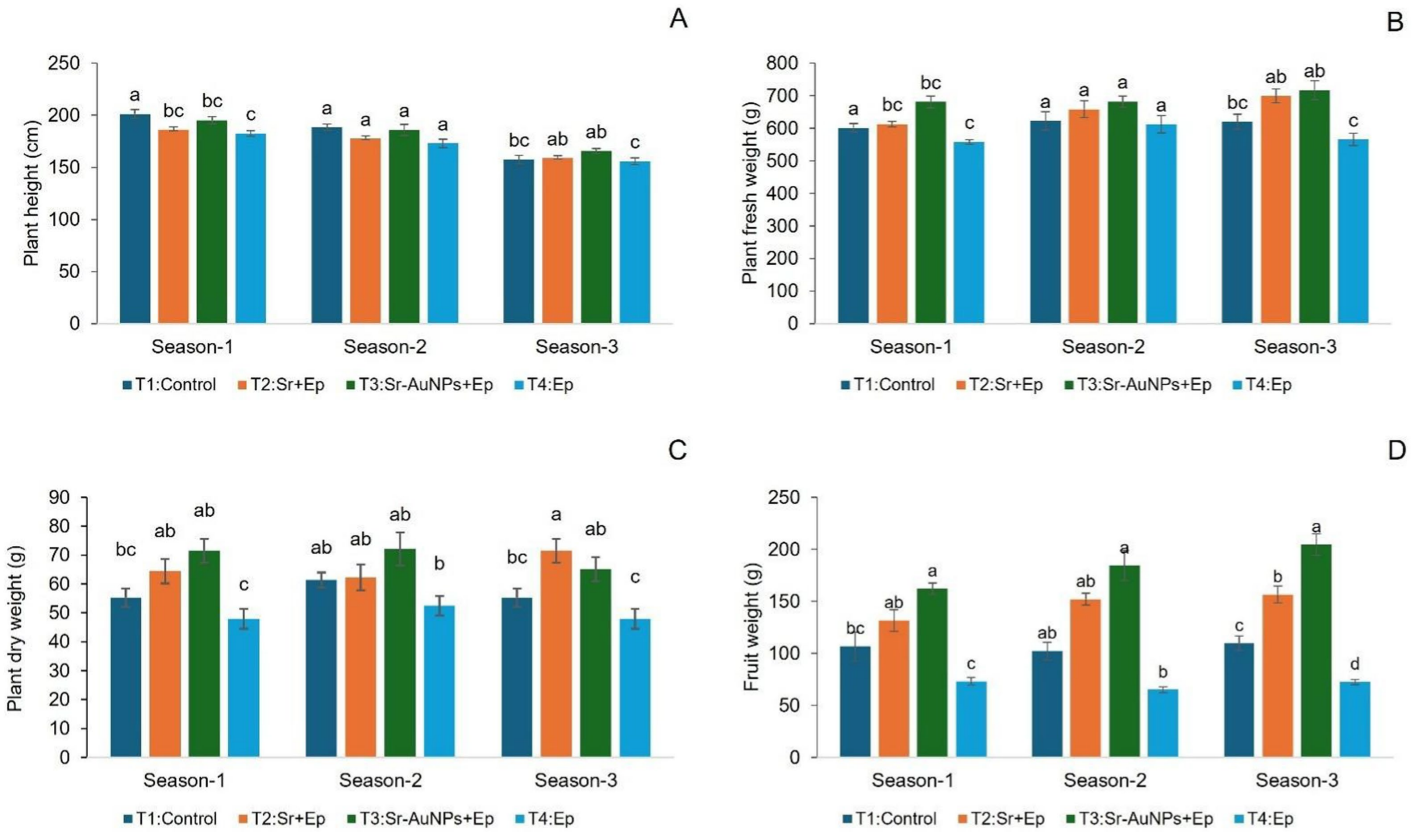
Comparative analysis of tomato plant height **(A)**, fresh weight **(B)**, dry weight **(C)**, and fruit weight **(D)** under different treatment regimens across three growing seasons. The data represent the means ± SDs (*n* = 20). Bars sharing the same letter within a group are not significantly different (*p* > 0.05) according to One-way ANOVA followed by a *post hoc* Tukey’s HSD test.

The effects of *S. rhizophila* (Sr) (T2) and AuNP-conjugated Sr. (T3) on tomato growth were evaluated across three consecutive greenhouse growing seasons by measuring plant height, fresh weight, dry weight, and fruit weight (Figures 4A–D).

The fruit weight’s responsiveness was the highest noticed among the three seasons shown in Figure 4D, with the use of AuNP-conjugated *S. rhizophila* (T3) having statistically increased total yield versus controls (T1, T4) and AuNP-unconjugated *S. rhizophila* (T2) (*p* < 0.05). In addition, statistically significant enhancements were also achieved in vegetative growth indices such as plant height (Figure 4A), fresh weight (Figure 4B), and dry weight (Figure 4C), but enhancements in these indices were much smaller relative to the increase in fruit biomass. The consistency of the results over three growing seasons indicates the bio-conjugate has great potential to act as a yield-stabilizing agent when grown in greenhouse conditions.

#### 3.4.1 Plant height

The height of plants differed significantly between each treatment as well as between growing seasons. In the first growing season, control plants (T1) had maximum height (201.35 ± 4.31 cm), which was statistically comparable to Sr-treated (T2) and Sr-AuNP-treated (T3) plants; however, T1 was taller than the pathogen-only plants (T4). In both Season 2 and Season 3, no significant differences were observed among T1, T2, and T3, while T4 consistently had the shortest height throughout both growing seasons, especially in Season 3 (155.80 ± 3.25 cm). In conclusion, the results indicate that plant height was significantly decreased by the pathogen challenge; however, treatments with bacteria mitigated this height reduction. Notably, there were no significant differences observed between the conjugated Sr-AuNPs (T3) and the Sr. (T2) treatments alone throughout all three growing seasons. This suggest that the bio-nanoparticle conjugation maintains the fundamental growth-promoting effects of the antagonist while enhancing stability and colonization, as demonstrated by our SEM and ICP-MS analyses.

This suggests that the bio-nanoparticle conjugation maintains the core growth-promoting effects of the antagonist while enhancing stability and colonization, as demonstrated by our SEM and ICP-MS analyses.

#### 3.4.2 Plant fresh weight

Fresh biomass accumulation showed treatment-dependent and season-specific responses. In Season 1, T3 plants displayed higher fresh weight (681.36 ± 18.23 g) compared with T1 and T4 and were comparable to T2. In Seasons 2 and 3, fresh weight increased across all treatments, with T2 and T3 consistently outperforming the pathogen-only treatment (T4). However, Tukey’s post-hoc analysis indicated no significant difference between T2 and T3 in Seasons 2 and 3, suggesting comparable effectiveness of PGPR alone and conjugated PGPR in sustaining vegetative biomass under pathogen pressure.

#### 3.4.3 Plant dry weight

Dry weight measurements reflected similar trends. In the first season, T2 and T3 averaged more than three times the dry weight of T4, with T3 having the highest value overall (71.50 ± 4.13 g). In the second and third seasons, plants with bacteria still had increased dry biomass than those with pathogens only (T4), yet the difference between T2 and T3 was not statistically significant, which suggests that their performance had overlapped during the later seasons.

#### 3.4.4 Fruit weight

Fruit yield was the parameter most consistently influenced by AuNP conjugation. Throughout the seasons, the T3 plants produced significantly higher fruit weight compared to other treatments, which increased from a mean of 162.00 ± 5.63 g in Season 1 to 204.62 ± 10.41 g in Season 3. Plants under T2 showed intermediate yields, significantly higher than T4 plants but lower than T3 plants, while infected only with the pathogen showed the lowest fruit weight. These results demonstrate a stable and reproducible enhancement of reproductive output associated with Sr–AuNP treatment (T3) across seasons.

### 3.5 Rhizobacterial population dynamics

**Figure 5:**
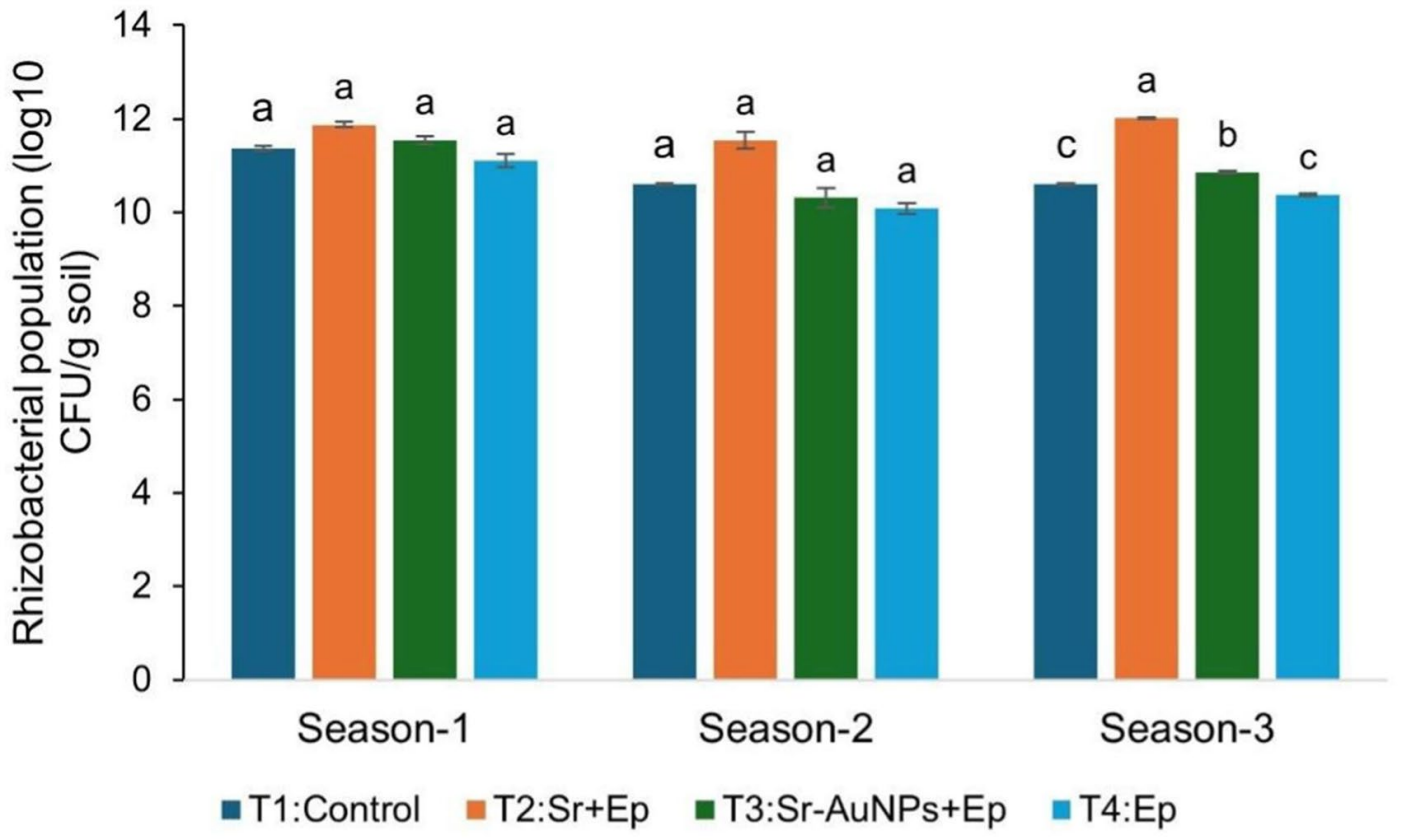
Rhizobacterial population dynamics in the tomato rhizosphere across treatment groups. Bars indicate the mean counts of total culturable rhizobacteria per gram of rhizosphere soil: (a) T1, Control, (b) T2, Sr. + Ep, (c) T3, Sr-AuNPs + Ep, and (d) T4, Ep. Error bars are calculated as SEM (*n* = 5). Bars sharing the same letter within a group are not significantly different (*p* > 0.05) according to one-way ANOVA followed by a *post hoc* Tukey’s HSD test.

Across three growing seasons, the population density of rhizobacterial populations within soil was evaluated for four treatment groups including T1 (control), T2 (Sr + Ep), T3 (Sr–AuNPs + Ep), and T4 (Ep only) as shown in Figure 5. Population density is represented by log₁₀ CFU per gram of soil. In Season 1, the statistical analysis showed no significant difference in the density of rhizobacteria between all treatment groups with an average of approximately 11.2 and 12.2 log₁₀ CFU/g soil (*p* > 0.05). In Season 2, T2 (Sr + Ep) had a substantially larger population density (~11.6 log₁₀ CFU/g) than T3 and T4 with a significant difference (*p* < 0.05). Average rhizobacterial population densities for T1 (control) were intermediate. By Season 3, treatment group populations expanded and exhibited greater differences between treatment groups. In terms of rhizobacterial density, T2 exhibited the greatest density (~12.0 log₁₀ CFU/g soil) compared to T1 and T4 (*p* < 0.05). T3 (Sr– AuNPs + Ep) had a moderate density compared to T1 and T4 but was still statistically significant (*p* < 0.05) in terms of density when compared to T2.

#### 3.5.1 Correlation with *Erwinia persicina* load

The impact of rhizospheric persistence of *S. rhizophila* on the abundance of the pathogen was evaluated by Spearman’s rank correlation analysis based on the treatment mean values of n = 4 in each season (Supplementary Table S3). Negative correlations were recorded across all three growing seasons, with *r*s values recorded at −0.2 and −0.4 in Seasons 1, 2, and 3, respectively. Coefficients of determination resulted in a range from 0.04 to 0.16, and covariance values were always negative between −0.33 and −0.67. This demonstrates that an inverse pattern between rhizobacterial persistence and pathogen load was recurrent. All these correlations values were not significant at *p* > 0.05, which may be due to using a limited number of treatments means in the correlation analysis. These results provide evidence that there exists a constant inverse relationship between *S. rhizophila* persistence and *E. persicina* colonization in favor of biocontrol action exerted by *S. rhizophila* in the rhizosphere.

### 3.6 Quantification of gold uptake in tomato root clusters

**Figure 6:**
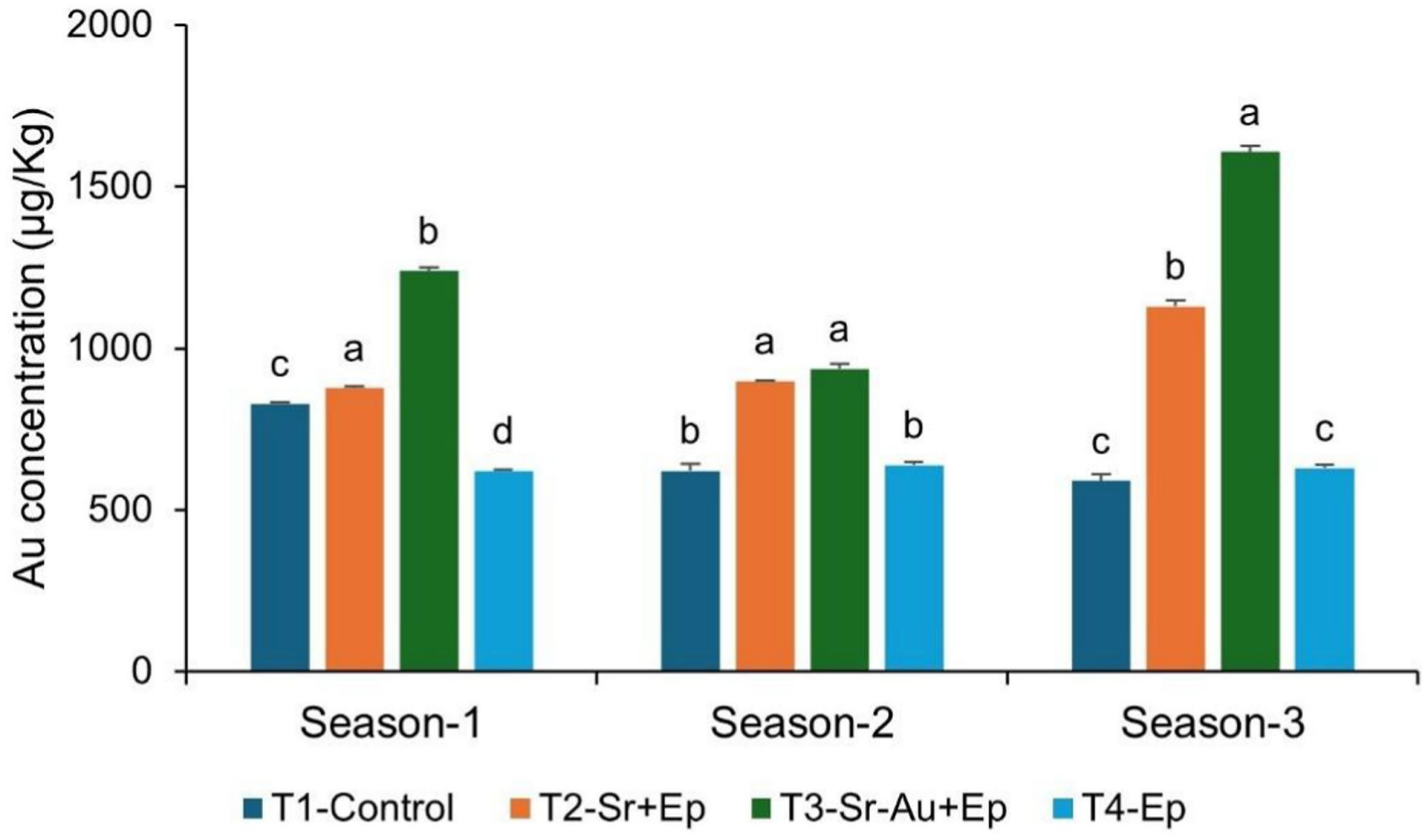
Gold nanoparticle (AuNP) accumulation in tomato root clusters across three growing seasons. The bars indicate the mean AuNP concentration in μg/kg for each treatment group, as determined by ICP-MS: (a) T1, Control, (b) T2, Sr. + Ep, (c) T3, Sr-AuNPs + Ep, and (d) T4, Ep. The values are presented as a function of season with error bars representing the standard error of the mean (SEM). Bars with similar letters indicate no significant difference in values for a growing season, as determined by One-way ANOVA with a Tukey’s HSD *post hoc* test, where *p* > 0.05.

The ICP–MS revealed maximum gold accumulation in T3 (Sr–AuNPs + Ep) roots 1238.70 ± 9.94 μg/kg (Season 1), 936.52 ± 15.17 μg/kg (Season 2), and 1,606.87 ± 20.81 μg/kg (Season 3) (Figure 6). Two-way ANOVA confirmed significant treatment, season, and interaction effects (*p* < 0.0001). This highlights the enhanced bioavailability and uptake of AuNPs delivered through bacterial conjugation.

### 3.7 FESEM–EDS localization of Sr. and Sr–AuNPs in tomato roots

**Figure 7:**
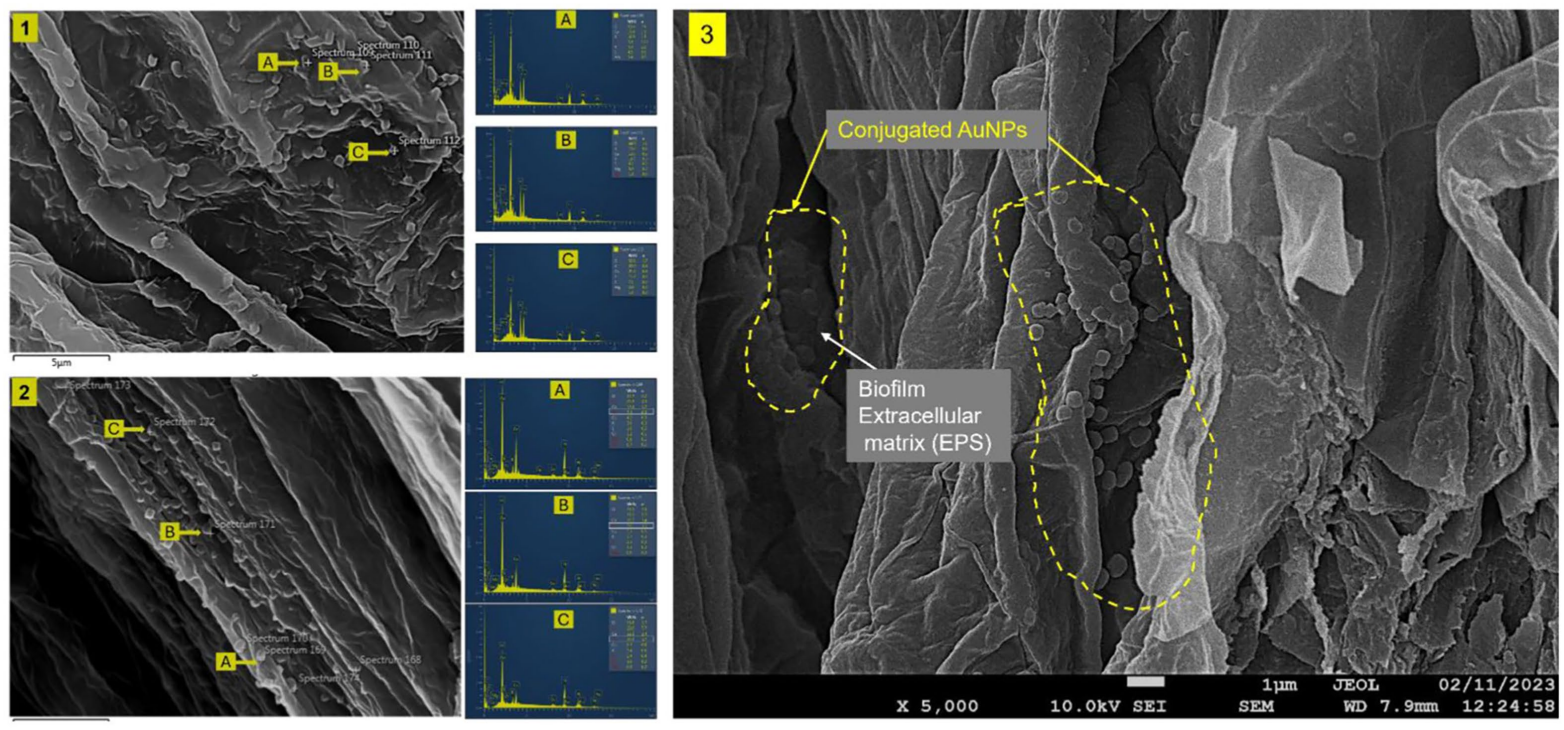
FESEM-EDS analysis of tomato root colonization. **(1)** FESEM image of T2 (Sr + Ep) showing rhizobacterial colonization without gold nanoparticles; insets (1A–1C) show EDS spectra confirming the absence of Au. **(2)** FESEM image of T3 (Sr-AuNPs+Ep) revealing extensive bacterial adherence and root integration; insets (2A–2C) show EDS spectra with clear Au peaks (7.1–10.2 wt.%) confirming nanoparticle conjugation. **(3)** High-resolution FESEM micrograph of T3 root surface illustrating dense bacterial biofilm formation with spherical AuNPs (indicated by clustered nanoscale particles) distributed along the root architecture. The nanoparticles appear closely associated with the extracellular matrix, supporting enhanced biofilm integration and nanoparticle retention. Scale bars: 5 μm (Panels 1, 2) and 1 μm (Panel 3). Magnification: 5000×.

FESEM images revealed dense bacterial colonization in both the Sr. [Figure 7(1)] and Sr–AuNP [Figure 7(2)] treatments. The EDS spectra confirmed that elemental Au (7.1–10.2 wt%) was present exclusively in the T3 roots, verifying that the AuNPs were delivered via bacterial conjugations. Other detected elements (O, C, Si, P, Ca, and S) indicated root tissue integrity and biofilm matrix composition. High-resolution FESEM imaging further revealed discrete, spherical nanoparticles densely distributed along the root surface and embedded within biofilm-like structures, indicating strong nanoparticle retention and association with the extracellular matrix (Figure 7(3)).

### 3.8 Symptom expression and disease severity index (DSI)

**Figure 8:**
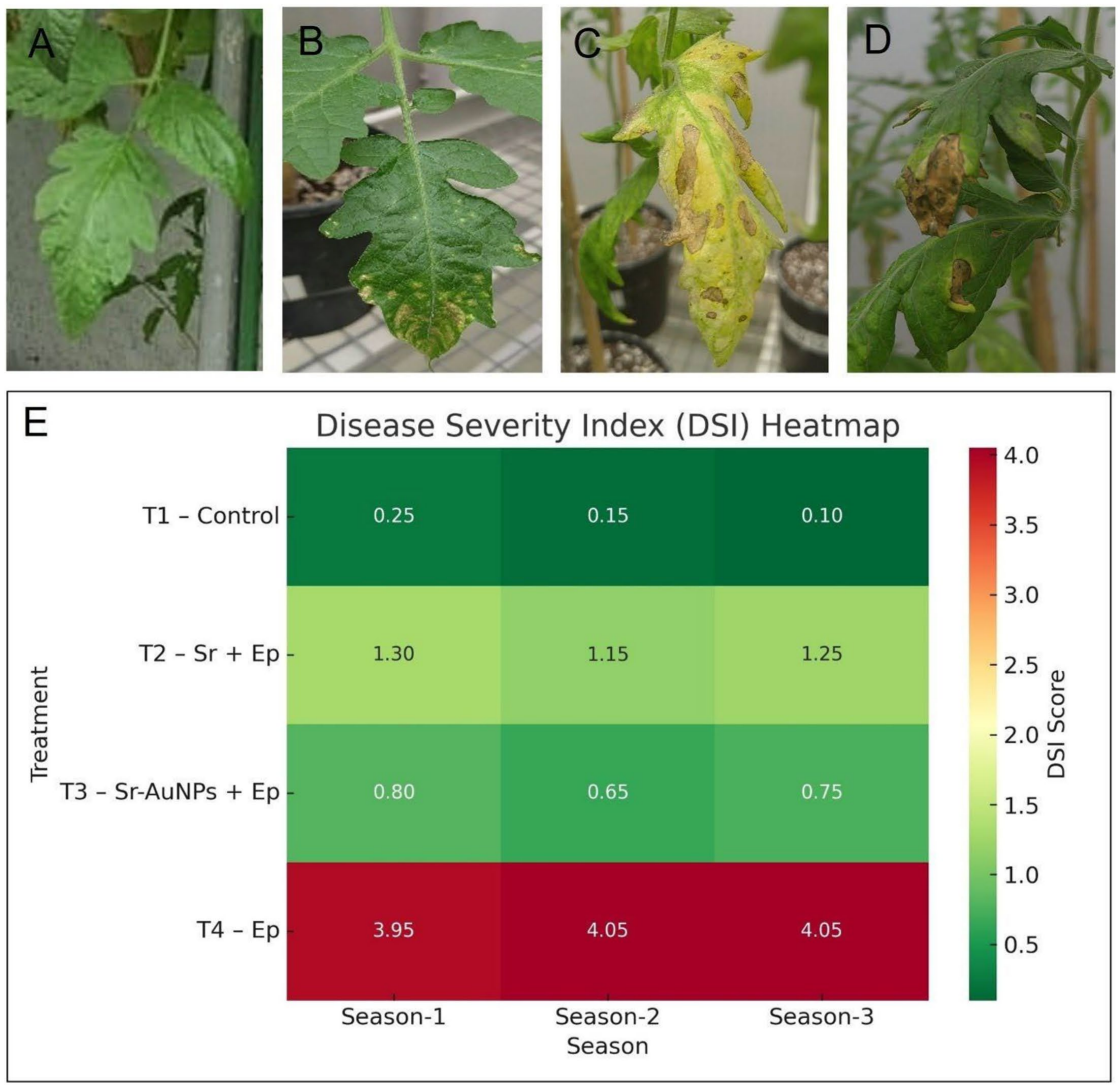
Pathological symptoms and disease severity in tomato plants. **(A–D)** Representative leaf symptoms illustrating the progression of *E. persicina* infection: **(A)** healthy leaf (T1), **(B)** early marginal chlorosis, **(C)** localized necrotic spots, and **(D)** advanced leaf blight with tissue collapse. **(E)** Heatmap of the Disease Severity Index (DSI) across three growing seasons. DSI scores range from 0.10 (healthy) to 4.05 (severe), demonstrating that the T3 treatment consistently maintains significantly lower severity levels than the T4 pathogen-only treatment.

The pathogenicity of *E. persicina* strain USTRW7 was confirmed through the observation of progressive leaf symptoms in greenhouse-grown tomato plants. Compared to the healthy appearance of control plants (Figure 8A), the early stages of infection were characterized by chlorotic lesions that originated at the leaf margins and tips (Figure 8B). As the disease progressed, symptoms developed into necrotic leaf tips (Figure 8E), localized necrotic spots (Figure 8C), and extensive leaf light (Figure 8D), which ended with complete tissue destruction in the treatment with the pathogen alone (T4). The changes in appearance were measured by a Disease Severity Index (DSI) and were found to be subject to significant variations that depended on the treatments during all three growing seasons (Figure 8E).

#### 3.8.1 Pathogen pressure

The plants in the T4 (Ep) group were the most affected by the disease throughout.

the experiment, recording DSI measures from 3.95 to 4.05, i.e., almost total leaf necrosis and deep infection.

#### 3.8.2 Biocontrol efficacy

The treatment of the plant with only *S. rhizophila* (T2) led to a decline in symptom intensity measured in the range of 1.15–1.30.

#### 3.8.3 Synergistic protection

The bio-conjugated treatment (T3: Sr-AuNPs+Ep) was the best among others that provided protection, for it kept its DSI scores (0.65–0.80) substantially lower than those of the T2 and T4 groups.

#### 3.8.4 Control baseline

The control (T1) plants did not show any symptoms with very low DSI scores (0.25). These phenotypic findings have a strong correspondence with the molecular detection of *E. persicina* DNA. The absence of symptoms in T3 coincides with the extremely low pathogen levels recorded in Season 1, when the AuNP-conjugated Sr. treatment was administered, marking its efficacy over the T2 and T4 groups.

### 3.9 Quantification of *Erwinia persicina* colonization in tomato leaves across growing seasons

**Figure 9:**
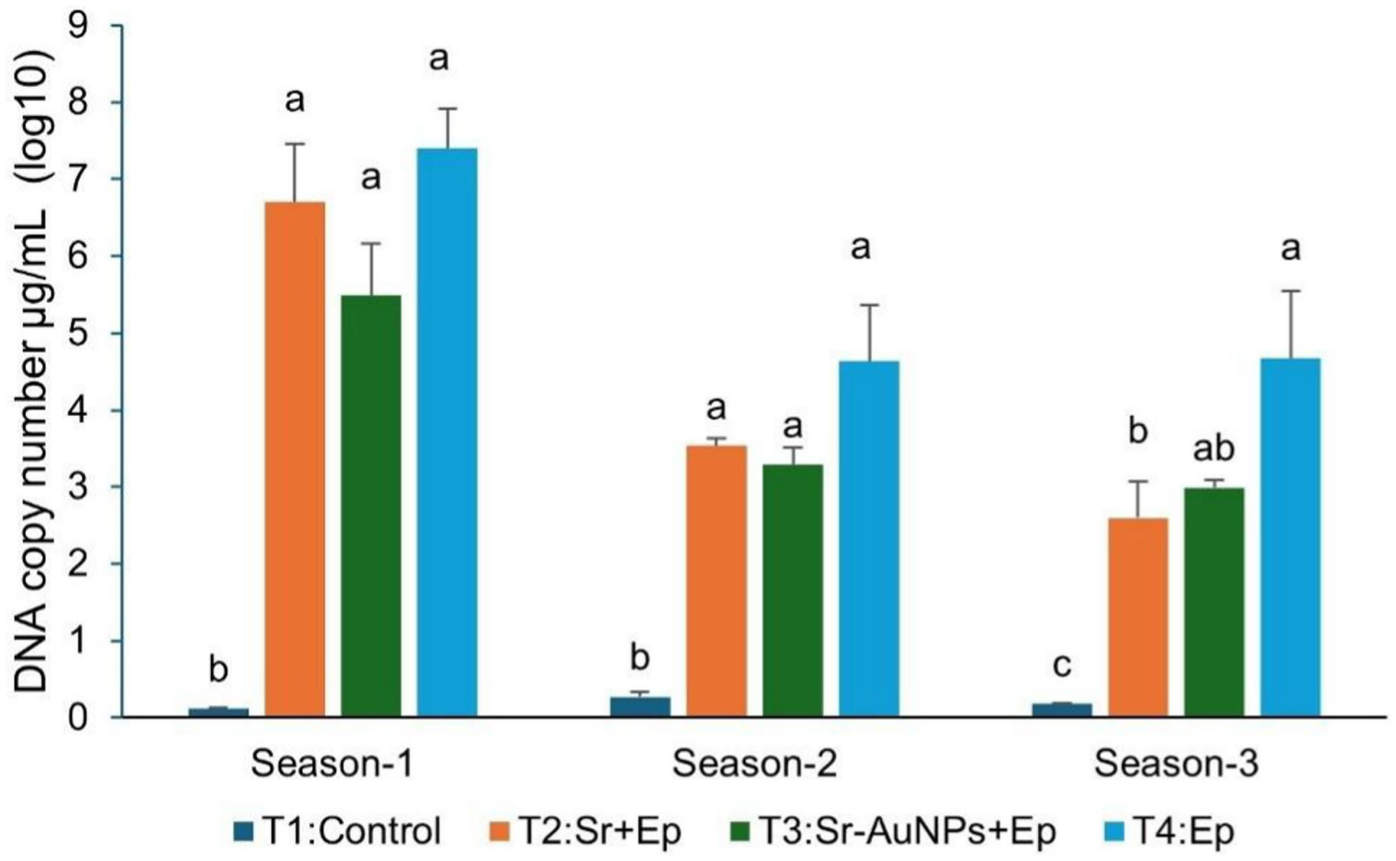
Seasonal quantification of *Erwinia persicina* DNA abundance in tomato leaves via RT–qPCR. Pathogen colonization levels are expressed as log10 DNA copy number per μL. Bars represent the mean DNA copy number per μL converted into log10 scale ± SEM (*n* = 8) measured across three growing seasons. Bars sharing the same letter within a group are not significantly different (*p* > 0.05) according to one-way ANOVA followed by a *post hoc* Tukey’s HSD test.

The RT-qPCR analysis showed a quantitative difference in the treatment of the *E. persicina* colonization in the tomato leaves through the three-season cycle (one-way ANOVA, *p* < 0.05; Figure 9). All three seasons, the highest *E. persicina* DNA copy numbers in the pathogen-only treatment (T4) confirmed the presence of the pathogen, and its proliferation through the plant’s systemically, while the control (T1) plants showed very little amplification, which means that there was no background contamination. In season 1, the AuNP-conjugated Sr. treatment (T3) greatly reduced the amount of the pathogen in comparison to both Sr. (T2) and pathogen-only (T4) treatments (Tukey’s HSD, *p* < 0.05). In the second and third seasons, both Sr. -treated groups (T2 and T3) had a significantly lower amount of the pathogen than T4, while the difference between T2 and T3 was not statistically significant.

These molecular trends were in line with the greenhouse phenotypic observations. In all seasons, the pathogen infection (T4) resulted in a significant decrease of plants’ height, biomass weight, and fruit yield in comparison to the control plants (*p* < 0.05). Sr. inoculation (T2) helped a little in making the negative effects less severe, while the AuNP-conjugated Sr. (T3) had consistently better growth results, especially in fruit weight, where T3 plants were claiming significant increases over all others in each season (Tukey’s HSD, *p* < 0.05). Importantly, the rank order of the treatments (the relative treatment rankings) did not change despite the absolute growth values varying from season to season, demonstrating that the results from these treatments were repeatable in a greenhouse environment.

## 4 Discussion

The current research demonstrates that the gold nanoparticles (AuNPs) that are biosynthesized from the marine green alga *C. sertularioides* could be successfully conjugated with the plant growth–promoting rhizobacterium *S. rhizophila* and used as a multifunctional bio-nanocomposite to improve tomato growth and prevention of *E. persicina* infection under greenhouse conditions. By integrating physicochemical characterization, microbial compatibility, plant growth responses, pathogen quantification, and elemental localization, the data provides insight into how biologically capped AuNPs influence plant–microbe–pathogen interactions.

### 4.1 Biosynthesized AuNPs exhibit structural integrity, stability, and biological capping

The UV–visible LSPR peak at 533 nm, together with XRD-confirmed FCC crystallinity, XPS verification of metallic Au^0^, and TEM-observed nanoscale morphology, collectively confirm the successful formation of structurally stable AuNPs. With a particle size range of 12.5 to 16.2 nm, the nanoparticles can interact with different kinds of biological entities in the most effective way possible; thus, not only the association with the surfaces of microbes and plants achieved, but also the efficiency of cytotoxicity is minimized. The balance between bioactivity and safety, as found in this system, has been well supported by recent publications. Even gold nanoparticles (AuNPs) in the size range of 10–20 nm have been found non-toxic at a concentration up to 100 μg/mL, without affecting the dry biomass or seed germination (Botteon et al., 2025; Niżnik et al., 2024; Zulfiqar et al., 2024). Venzhik et al. (2021) reported that when employed in microdoses up to 100 μg/mL, AuNPs are typically non-toxic and can even act as “adaptogens,” improving plant resistance to stress and increasing chlorophyll activity. These results, along with similar results found in silver nanoparticles (Alfosea-Simón et al., 2025) and nanocomposites derived from plants (Ditu et al., 2024; Chukwukwe et al., 2025), support our visual as well as yield-based results pointing toward the lack of phytotoxicity.

Both FTIR and TGA spectra distinctly revealed the participation of algal biomolecules like polysaccharides, proteins, and phenolics in nanoparticle reduction and stabilization. Biogenic capping agents significantly enhance nanoparticle properties by improving colloidal stability, preventing aggregation, and increasing biocompatibility compared to chemically synthesized counterparts (Ovais et al., 2018; Javed et al., 2020; Ghosh et al., 2021). The present study’s slightly negative zeta potential value (−16.4 mV) supports electrostatic stabilization of the colloidal suspension, revealing adequate repulsive forces to impede the agglomeration of particles (Kłodzińska et al., 2010). This observation agrees with the finding that plant-extracted gold nanoparticles with surface charges that are moderately negative would be strong in colloidal stability and have good biological compatibility (Koutsoulas et al., 2012; Holišová et al., 2021; Elhabal et al., 2022; Alabdallah, 2025). A negatively charged surface potential, to the extent of moderation, is advantageous for bacterial binding because regions of microbial cell walls that are negatively charged can engage through van der Waals and hydrogen-bonding interactions (Wilson et al., 2001; Pajerski et al., 2019; Wilhelm et al., 2021). The three mentioned features: biogenic surface functionalization, colloidal stability, and optimized charge characteristics combine to make the *C. sertularioides*-derived AuNPs a perfect match for microbial conjugation and agricultural applications, where it is critical to keep the environment safe and maintain biological compatibility (Kalantari and Turner, 2024; Arcos Rosero et al., 2024).

Biocompatibility and growth response of *Stenotrophomonas rhizophila* to AuNPs *S. rhizophila* showed a clear concentration-dependent response to AuNPs (Figure 2) during the growth phase. Non-toxic and slightly growth-promoting were the concentrations of 100 and 400 μg/mL, whereas the 500 μg/mL and above ones caused gradual inhibition of growth but did not entirely shut down bacterial reproduction, thus a bacteriostatic effect was observed at the higher doses. Most importantly, the 100 μg/mL concentration applied in experiments with plants was the same as the biologically compatible one.

The dose-dependent reduction in cellular growth is consistent with previous findings that demonstrate that exposure to high concentrations of gold nanoparticles can negatively impact developmental processes, including oxidative stress (i.e., a build-up of harmful reactive oxygen species), perturbation of cellular membranes (mortality), and disruption of DNA replication processes (Shukla et al., 2015; Gupta et al., 2016; Shaikh et al., 2019; Milewska-Hendel et al., 2019; Linklater et al., 2020). Although exposure of a bacterial cell to high concentrations of gold nanoparticles may not completely eliminate cell viability, decreased cellular growth is likely due to increased nanoparticle-cell interactions and a corresponding increase in the intracellular stress level of the cell, as opposed to a single acute toxic effect.

It is noteworthy that while numerous gold nanoparticle concentrations were evaluated *in vitro*, only one concentration (100 μg/mL gold nanoparticles) was tested on *S. rhizophila* for the purpose of conjugation. Based on the data presented in Figure 2, this is the concentration that produced the most favorable profile for growth and represents a suitable concentration to use for future studies involving downstream applications. While 100 μg/mL of freely dispersed gold nanoparticles may be high concentration, the physicochemical properties and biological behavior of the gold nanoparticles were fundamentally altered when they became conjugated to the surface of a bacterial cell. TEM, DLS, and FTIR studies revealed successful attachment of AuNPs to bacterial cell surfaces without damaging the morphology, with considerable decrease in their hydrodynamic size after conjugate formation (Figures 3A–F). Biological immobilization by such nanoparticles has been well demonstrated to minimize aggregation and maximize the number of reactive nanoparticles in direct contact with the root systems of plants, hence preventing oxidative stress in plants (Compant et al., 2019; Trivedi et al., 2020). Moreover, nanoparticles linked with PGPR remain entrapped in biofilm/EPS matrixes, thereby controlling nanoparticle reactivity in the rhizosphere region around plants (Flemming and Wuertz, 2019; Bhatia et al., 2021).

Though classical biomarker measurements of phytotoxicity (e.g., chlorophyll concentration, electrolyte leakage, and malondialdehyde content) were not performed, the lack of indication of stress symptoms and the consistent enhancement of plant height, biomass, and yield across three growing seasons provide integrative evidence against chronic phytotoxicity (Thakur et al., 2018). Oxidative stress caused by nanoparticles in plants has been often reported to negatively impact plant growth and fertility (Tripathi et al., 2017; Rizwan et al., 2015; Gowtham et al., 2024), and none of these effects were noted in treatment T3. Moreover, natural capping of AuNPs biosynthesized can supposedly enhance their biocompatibility (Gurunathan et al., 2014; Shaikh et al., 2019). Overall, these findings confirm that AuNP–*S. rhizophila* conjugates could be safely used to enhance plant growth at chosen concentrations: however, the biochemical stress responses must be analyzed in further research.

### 4.2 Concentration-dependent bacterial compatibility governs effective conjugation

The biphasic growth response of *S. rhizophila* to AuNPs fits a broader pattern where metallic nanoparticles can be mildly stimulatory at low doses but disrupt cellular homeostasis and growth at higher doses, via combined effects on membranes, redox balance, and metabolism (Slavin et al., 2017; Wahab et al., 2023). Several reports indicate that the bacterial responses to metal/metal oxide nanoparticles were concentration-dependent and occurred in two phases, which is in line with our observation for *S. rhizophila.* Iron oxide NPs caused the growth of *Enterococcus hirae* to be stimulated at low concentrations and to be inhibited at higher ones, while the effect on *E. coli* was mainly inhibition; thus, both species and dose have an effect (Gabrielyan et al., 2019). The presence of metallic NPs (Cu, Zn, CuO, ZnO) in the leachate microbiota resulted in a reduction of bacterial growth and a decrease in the quantity of antibiotic resistance genes in a dose-dependent fashion, the mechanisms of which included growth inhibition, ion dissolution, and oxidative stress (Bresee et al., 2011; Su et al., 2019).

The compatibility window explored in the current study allowed successful conjugation without affecting bacterial viability. Several studies indicate that optimizing the size, surface charge, and concentration of AuNPs will allow for better interaction between AuNPs and bacteria while also preserving cell integrity and morphology (Zhu et al., 2012). When AuNPs are created that are either moderately or biologically functionalized they may be conjugated into various biomolecules (i.e., nucleic acids, proteins, and antibiotics), allowing the AuNPs to interact with living cells without significant levels of toxicity at optimal concentrations (Pourali et al., 2021; Pourali et al., 2023).

TEM and DLS observations clearly revealed strong bindings of AuNPs to the bacterial surface without significant morphological changes, implying that the binding was limited to the bacterial surface rather than entering the bacterial cells or damaging the bacterial membranes (Elliott et al., 2021; Pourali et al., 2021; Pourali et al., 2023).

The FTIR identification of Au–N and Au–O bonding correlations, in line with previous reports on bio-nanoconjugates, indicates the formation of stable metal–ligand interactions between bacterial protein and polysaccharide functional groups and the gold nanoparticle surface (Li et al., 2016; Sayed and El-Sayed, 2019; Pourali et al., 2023; Nagoth et al., 2024). FTIR investigations of metal and metal-oxide nanoparticles in most cases reveal that proteins and polysaccharides have amine, carboxyl, hydroxyl, and phosphate groups that bond to the nanoparticle surfaces, thus acting as capping and stabilizing ligands (Li et al., 2016; Sayed and El-Sayed, 2019; Pourali et al., 2023; Nagoth et al., 2024; Pasieczna-Patkowska et al., 2025). In a similar way, shifts and the emergence of new bands in the amide, O–H/N–H, and C–O areas have been used to verify the presence of nanoparticles in bacterial biomolecules and biogenic capping layers.

### 4.3 AuNP conjugation enhances functional performance without impairing rhizosphere competence

Over a period of three growing seasons, both the PGPR treatments (Sr, and Sr-AuNPs) were able to counter the inhibited growth caused by the pathogen, especially in terms of biomass production and plant height. Non-significant variabilities between T2 and T3 for each vegetative parameter indicate there was no decrement in the natural plant growth promotion properties of *S. rhizophila* by AuNP conjugation. Additionally, rhizobacterial population changes revealed a stable rhizosphere presence for all treatments in all three seasons, thereby establishing the non-hazardous effect of nanoparticle conjugation on bacterial populations in soil.

The variation in the bacterial counts across the seasons could be attributed to environmental fluctuations characteristic of greenhouses. Interestingly, it was observed that during Season 3, the highest populations of rhizobacteria were sustained in treatment inoculated with Sr. (T2) alone, whereas moderate populations were sustained in treatment AuNP-conjugated bacteria (T3). Such decline could be attributed to minute changes associated with root attachment efficiency, surface properties, or competitiveness within the rhizosphere following surface modification or exposure to nanoparticles, as it has been previously documented that exposure or surface modification with nanoparticles alters growth, colonization properties, or associated interactions within the PGPR colonization system (Kong and Liu, 2022; Verma et al., 2024).

### 4.4 Enhanced root uptake of gold nanoparticles via AuNP-conjugated *Stenotrophomonas rhizophila*

The ICP-MS quantification indicated that significantly more gold had accumulated in the tomato root clusters treated with the *S. rhizophila*–AuNP conjugate along with *E. persicina* (T3) throughout the three growing seasons (Figure 6). The gold concentration in T3 roots was between 936.52 ± 15.17 μg/kg and 1606.87 ± 20.81 μg/kg, which was significantly higher than that in all other treatments (*p* < 0.05). The increase seen in the amount of gold in the T3 plants supports the hypothesis that *S. rhizophila* facilitates the availability of nanoparticles in the rhizosphere through the formation of biofilms, the production of EPS, and close contact surfaces on the roots. Biofilms, and EPS, have been shown to stabilize nanoparticles, prevent their agglomeration, and concentrate their retention at biological surfaces (Flemming and Wuertz, 2019; Singh et al., 2022). Mendes et al. (2013) and Compant et al. (2019) further supported the idea that an established PGPR-colonization creates an area of residency, or micro-niches, at the root–soil interface for providing a stable location for the persistence of microbial life, and a location of localized biochemical action to facilitate the transport of compounds associated with PGPR. Similarly, Palmqvist et al. (2015), Trivedi et al. (2020) and Bhatia et al. (2021) showed that communities of microbes associated with a plant improve their functional availability in the rhizosphere, through mediating the transport of, stabilization of, and interaction between plant roots and external materials.

From the current study, the significant decrease in hydrodynamic size following conjugation, and as well as the confinement of Au presence to T3 roots, provides evidence that *S. rhizophila* functioned as a biological delivery vehicle for AuNPs. Recent research has also identified that interactions between PGPR and nanoparticles play a significant part in the enhancement of rhizosphere engineering and improving plant growth (Alzate Zuluaga et al., 2024; Zaman et al., 2025). All findings indicate that T3 plant growth and protection arise through a “synergistic” nano-bio mechanism, with AuNPs functioning mainly as support and transporter to enhance efficiency and capacity of colonizing and delivering PGPRs to the plant, rather than being separate antifungal agents. Moreover, FESEM images and EDS analysis have drawn a similar inference. The roots of T3, plants exhibited high amounts of bacterial colonization with obvious gold signals (7.1–10.2 wt.%) which were present not only on the root surfaces but also on the bacterial biofilms (Figure 7, Panel 2). On the other hand, the roots treated with bacteria alone (T2) were found to be colonized but not with any signs of gold peaks (Figure 7, Panel 1), verifying that AuNPs had penetrated through bacterial conjugates. The observed clustering of nanoparticles within the biofilm matrix further supports the reduced hydrodynamic size observed in DLS, suggesting a compact conjugate architecture that enhances surface adherence and facilitates closer interaction with root tissues (Figure 7, Panel 3). The presence of AuNPs among the bacterial biofilm’s hints at a combined action in aiding root colonization and persistence, thus interacting more efficiently with root epidermal cells (Kłodzińska et al., 2013; Malejko et al., 2021). The high IAA production potential of *S. rhizophila* GSB-381, measured at 118.61 μg/mL, may contribute to the large degree of root surface remodeling observed in the SEM results [Figure 7(1)]. The localized production of IAA by the bacteria embedded in the root structures [as evidenced by the 5,000× magnification Figure 7(3)] allows for root hair elongation, increasing the root surface area for further colonization and growth stabilization by the AuNPs.

The SEM analysis confirmed that the AuNP-*S. rhizophila* conjugate does not merely sit on the surface but exhibits intimate nesting [indicated by the dotted yellow circles in Figure 7(3)] within the root micro-structures. This close-range interaction, supported by the integration of cells into the biofilm extracellular matrix (EPS), aligns with DLS data showing a smaller hydrodynamic size, which likely reduces steric hindrance and promotes deeper tissue association.

Tissue-specific quantification of AuNPs in roots provides important mechanistic insight into the observed growth promotion and pathogen protection (Avellan et al., 2017; Milewska-Hendel et al., 2019; Ferrari et al., 2021; Xue et al., 2025). The present study has been focused on root tissues where PGPR colonization and nanoparticle delivery are most intense. Further investigation of leaves and fruits would be advantageous to clarify systematic translocation and its possible effect on *E. persicina* colonization in aerial tissues. Such differentiation is crucial since differing distributions of nanoparticles can affect the efficacy of biocontrol and plant physiological parameters.

The comprehensive characterization of the AuNPs (through TEM, SEM–EDS, XRD, XPS, zeta potential, and TGA) confirmed that they had stable and good conjugation with *S. rhizophila*, which reduced the hydrodynamic size and limited the aggregation. These nano–bio conjugates possibly prolong the retention time in the rhizosphere and, therefore, the delivery system of bacteria is more efficient than applying free nanoparticles (Kah et al., 2018; Rai et al., 2018). Besides, the integration of AuNPs in the bacterial biofilms could be a way of sheltering both the microbes and the nanoparticles from the environmental stresses thus facilitating the success of colonization and functional interactions at the root surface. Overall, these results suggest that PGPR conjugation is a powerful technique for delivering nanoparticles to the plant roots, resulting in higher uptake that is accompanied by improved growth and resistance to diseases. The future implementation of tissue-based quantification of nanoparticles beyond roots will help clarify the nanoparticle distribution and its implication for the overall health of the plant.

### 4.5 AuNP conjugation preferentially enhances reproductive output and pathogen suppression

The treatment with Sr-AuNPs (T3) showed a marked and significant augmentation in the fruit weight values in all the growing seasons among the plant parameters tested (Figure 4). The trend indicates that the conjugation effect of gold nanoparticles might be more influential on the reproductive functioning rather than the vegetative functions. The augmentation in the fruit production might be correlated with the regulation of the pivotal functions of the nanoparticle, such as the regulation of nutrient assimilation, redox regulation, and phytohormonal regulation, which play pivotal roles in the development of the reproductive structures. Earlier studies have revealed that the augmentation of the antioxidant potential, especially the balance of the phytohormones such as auxins, gibberellins, and cytokinins, via nanoparticle treatment can create favorable conditions for the development of flowers and fruits, as it safeguards against the oxidative stress prevailing during the reproductive phase (Egamberdieva et al., 2017; Thakur et al., 2018; Su et al., 2019; Daniel et al., 2024; Ni et al., 2024). Thus, the findings of the experiments signify the potential of the Sr-AuNPs in enhancing reproductive efficiency through the cumulative effect of the regulation of the functions of nutrients, redox, and phytohormones.

### 4.6 Mechanistic synergy and seasonal robustness of *Stenotrophomonas rhizophila*–AuNP conjugates in suppressing *Erwinia persicina*

RT-qPCR–based quantification of *E. persicina* DNA provided strong molecular validation of the phenotypic disease suppression observed in greenhouse trials. The consistently higher pathogen load in T4 plants across seasons confirms aggressive systemic colonization in the absence of biocontrol agents. Both PGPR treatments significantly reduced pathogen abundance, with AuNP-conjugated *S. rhizophila* exhibiting superior suppression in the first season. This enhanced early-season performance may reflect a synergistic effect between PGPR-mediated competition and nanoparticle-induced antimicrobial pressure. Antibacterial modes of action attributed to gold nanoparticles include the disruption of biofilm development and cell surface interactions, such as electrostatic disruption of pathogenic adherence, and inhibition of virulence-regulating quorum sensing systems (Yu et al., 2016; Qais et al., 2021; James and Kumar, 2022; Lee et al., 2021).

Metabolic profiling studies have shown that exposure to AuNP disturbs central bacterial metabolism, including energy production, protein synthesis, and communication pathways that, in total, compromise bacterial viability (Chen et al., 2025). Functionalized AuNPs enhance such activities through the elevation of intracellular reactive oxygen species and the direct interference with quorum-sensing signaling in Gram-negative bacteria (Wang et al., 2023).

Indeed, gold nanoparticles have been reported to disrupt bacterial membranes, interfere with quorum sensing, and induce oxidative stress in phytopathogens-especially when their delivery is brought closer by microbial carriers (Ma et al., 2015; Slavin et al., 2017; Marques et al., 2023; Chen et al., 2025). While differences between T2 and T3 diminished in the later seasons, the consistent ranking of treatments is a testament to the robustness and reproducibility of the bio-nanocomposite strategy.

### 4.7 Integration of microbial dynamics and biocontrol mechanism

The combined analysis of the dynamics of rhizobacterial population and the level of pathogen helps in understanding the observed biocontrol efficiency in this study. CFU analysis revealed that the number of *S. rhizophila* remained significantly higher in treatment T2 and T3 than in the pathogen control, with AuNP-conjugated *S. rhizophila* (Treatment T3) maintaining the most consistent level. At the same time, RT-qPCR analysis revealed that the level of *E. persicina* DNA decreased substantially in those samples.

This negative correlation strongly suggests the existence of biocontrol through competition exerted by microorganisms at the root-soil interface. Continuous colonization by *S. rhizophila* would prevent the establishment of pathogens through competition for nutrients, binding sites, and rhizosphere space. This competition has been well-documented as the primary mechanism contributing to the biocontrol of diseases through the use of efficient PGPR, particularly when these pathogens target bacteria associated with soil and roots.

In particular, the increased suppression of pathogens in the initial stages of growth, as measured in T3, was associated with greater numbers of *S. rhizophila* and lesser numbers of *E. persicina*, indicating that the AuNP conjugation increased the ability of the bacteria to persist and compete effectively in the critical initial stages of plant establishment. This could be due to the increased ability of the nano-conjugate to bind to plant roots and the soil, thereby increasing the pressure exerted on the pathogen through competition. Additionally, the presence of the rhizobacterial system could contribute to the induction of systemic tolerance, as a result of prolonged interaction with plant roots.

Even though the differences in T2 and T3 reduced over the course of the later seasons, the presence of the treatment with increased *S. rhizophila* persistence correlating with reduced pathogen counts illustrates the robustness of the biocontrol relationship. The data described together indicate the consequence of the bio-nanocomposite approach, where the underlying factor for this effectiveness is the ability to facilitate rhizobacterial colonization, which once again affects pathogen proliferation through a possible plant defense response.

### 4.8 Implications for sustainable disease management

Collectively, these findings position Sr–AuNP conjugates as a promising, eco-friendly alternative to conventional chemical disease control strategies. The eco-friendly compatibility of PGPR (Upadhayay et al., 2023; Khoso et al., 2024) and the functional versatility of biogenic nanoparticles make it possible to implement a method that works toward goals of sustainability and precision agriculture (Backer et al., 2018; Parisi et al., 2015; Sashidhar et al., 2019; Sodhi et al., 2025). Importantly, the use of algal-derived AuNPs further enhances environmental safety by avoiding toxic reducing agents and ensuring biological capping. Future studies should explore transcriptomic and metabolomic responses of both host plants and pathogens to elucidate the signaling pathways underlying the observed growth promotion and disease suppression. Field-scale validation will also be essential to confirm the scalability and long-term ecological impact of PGPR– nanoparticle conjugates.

## 5 Conclusion

The findings of this study demonstrated that gold nanoparticles (AuNPs) generated from *Caulerpa sertularioides* extract can be successfully conjugated to the plant growth-promoting rhizobacterium *Stenotrophomonas rhizophila* to improve its ability to colonize tomato plants and to limit disease caused by *Erwinia persicina*. Several physicochemical and ultrastructure analyses confirmed that the AuNPs were stable in terms of dispersion, uniform in size, and successfully conjugated to the bacterial surface. Compared with the non-conjugated (T2) and control (T4) groups, the AuNP-conjugated *S. rhizophila* (T3) group presented improved plant growth parameters, reduced disease severity, and decreased pathogen DNA loads in host tissues over three consecutive cropping seasons. Overall, the results from our work demonstrated that nanoparticle-based microbial inoculants can provide more sustainable and ecologically friendly solutions to improve plant health, reduce stress under biotic challenges and directly affect climate change. The use of green nanotechnology in conjunction with microbial biocontrol agents offers the potential for precision agriculture and agricultural practices that can be more environmentally sustainable.

## Data Availability

The *Stenotrophomonas rhizophila* strain sequence used in this study was submitted to the NCBI GenBank database under accession number MK161197. All other data supporting the conclusions of this article, including the FESEM-EDS characterization and greenhouse trial results, are included within the article or its Supplementary material. Further inquiries can be directed to the corresponding author.

## Footnotes

1
https://astatsa.com/
