# Supplementary material for: Biogenic gold nanoparticles conjugated with rhizobacteria enhance tomato growth and suppress pathogen infection
Source: Front Microbiol. 2026 May 5;17:1758150. doi: 10.3389/fmicb.2026.1758150 (PMC13183646; doi:10.3389/fmicb.2026.1758150)
Supplement: Supplementary file 1 [file Supplementary_File_1.docx]

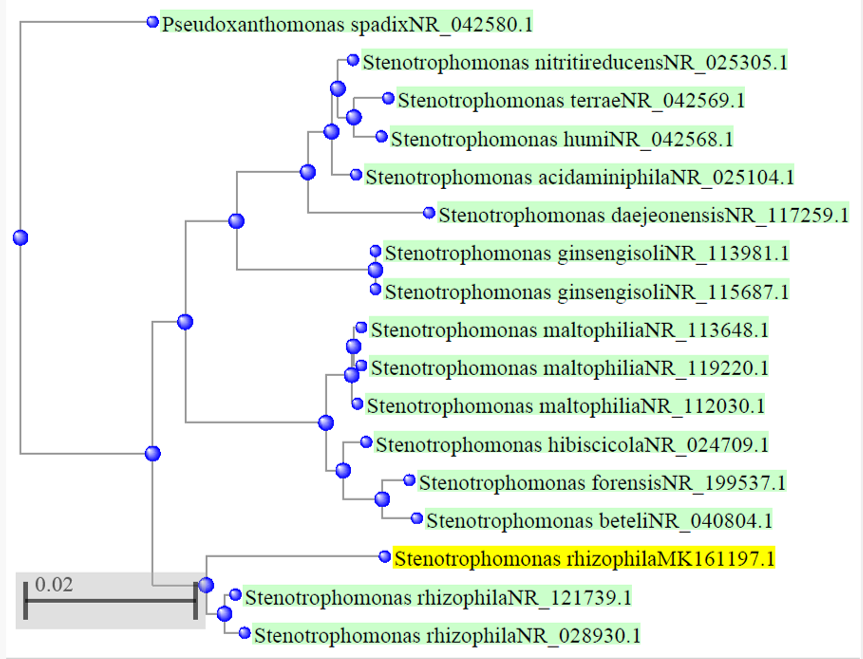


**Figure S1. Phylogenetic placement of** *Stenotrophomonas rhizophila* GSB-381 (**MK161197.1.)** The distance-based tree (Neighbor joining tree) shows the isolate (yellow) clustering with *Stenotrophomonas rhizophila* reference strains (NR_121739.1, NR_028930.1). *Pseudoxanthomonas spadix* serves as the outgroup. The scale bar represents 0.02 substitutions per site, confirming high genetic homology within the *S. rhizophila* clade and clear divergence from *S. maltophilia*.
