# Supplementary material for: Biogenic gold nanoparticles conjugated with rhizobacteria enhance tomato growth and suppress pathogen infection
Source: Front Microbiol. 2026 May 5;17:1758150. doi: 10.3389/fmicb.2026.1758150 (PMC13183646; doi:10.3389/fmicb.2026.1758150)
Supplement: Supplementary file 2 [file Table_1.docx]

**Table S1. (A)** Detailed morphological, biochemical, and Plant Growth-Promoting (PGP) Traits of *Stenotrophomonas rhizophila* GSB-381.

| **Category** | **Characteristic** | **Result / Observation** |
| --- | --- | --- |
| Identification | NCBI Accession No. | MK161197.1 |
|  | Isolation Source | Rhizosphere soil (Faba beans) |
| Morphology | Gram Staining | Negative (-) |
|  | Cell Shape | Rods |
|  | Motility / Capsule | Positive (+) |
|  | Spore Formation | Negative (-) |
| *Biochemical | Oxidase / Catalase | Positive (+) |
|  | Citrate Utilization | Positive (+) |
|  | Methyl Red / VP | Negative (-) / Positive (+) |
|  | H₂S Production | Positive (+) |
|  | Gelatin Liquefaction | Positive (+) |
| *Carbohydrate Utilization | Glucose / Lactose | Positive (+) / Positive (+) |
|  | Maltose / Trehalose | Positive (+) / Positive (+) |
| # PGP Traits (Quantitative) Indole acetic acid (IAA) production assay | IAA (0.15g/100ml Trp) | 118.61 ± 18.6 µg/mL |
|  | IAA (Basal Trp) | 26.07 ± 3.0 µg/mL |

**Note:** *Biochemical and Carbohydrate Utilization tests using API20E strip. # PGP Traits (Quantitative) Indole acetic acid (IAA) production assay by Salkowski colorimetric assay.

**Table S1.** (B) Strain-specific calibration of optical density (OD_600_ = 0.1) to viable cell counts (CFU/mL).

| **Bacterium** | **Mean CFU (10^5^ dilution)** | **Mean CFU**  **(10^6^ dilution)** | **Final Estimated Concentration (CFU/mL)** |
| --- | --- | --- | --- |
| *Erwinia persicina* | 160.2 ± 28.1 | 31.5 ± 8.7 | ≈2.4 × 10^8^ |
| *Stenotrophomonas rhizophila* | 481.9 ± 31.7 | 64.5 ± 11.3 | ≈5.6 × 10^8^ |

Note: CFU/mL values were calculated based on the means of biological replicates (n=3) using a 100 µL plating volume. These values were used to standardize all experimental inocula to a final concentration of ≈10^8^ CFU/mL.
