# Supplementary material for: Biogenic gold nanoparticles conjugated with rhizobacteria enhance tomato growth and suppress pathogen infection
Source: Front Microbiol. 2026 May 5;17:1758150. doi: 10.3389/fmicb.2026.1758150 (PMC13183646; doi:10.3389/fmicb.2026.1758150)
Supplement: Supplementary file 3 [file Table_2.docx]

Table S2. Primer pairs designed for *Erwinia persicina* strain USTRW7 (GenBank accession KU923347.1). Only primer pair 3 was used in RT‑PCR.

**Primer pair 1**

|  | **Sequence (5'->3')** | **Template strand** | **Length** | **Start** | **Stop** | **Tm** | **GC%** | **Self complementarity** | **Self 3' complementarity** |
| --- | --- | --- | --- | --- | --- | --- | --- | --- | --- |
| **Forward primer** | AAGAAGCACCGGCTAACTCC | Plus | 20 | 120 | 139 | 60.04 | 55.00 | 4.00 | 0.00 |
| **Reverse primer** | AGCGTCAGTCTTTGTCCAGG | Minus | 20 | 382 | 363 | 59.97 | 55.00 | 3.00 | 3.00 |
| **Product length** | 263 | | | | | | | | |

**Primer pair 2**

|  | **Sequence (5'->3')** | **Template strand** | **Length** | **Start** | **Stop** | **Tm** | **GC%** | **Self complementarity** | **Self 3' complementarity** |
| --- | --- | --- | --- | --- | --- | --- | --- | --- | --- |
| **Forward primer** | CCTGGACAAAGACTGACGCT | Plus | 20 | 363 | 382 | 59.97 | 55.00 | 3.00 | 1.00 |
| **Reverse primer** | GTTTACGGCGTGGACTACCA | Minus | 20 | 442 | 423 | 60.04 | 55.00 | 5.00 | 3.00 |
| **Product length** | 80 | | | | | | | | |

**Primer pair 3**

|  | **Sequence (5'->3')** | **Template strand** | **Length** | **Start** | **Stop** | **Tm** | **GC%** | **Self complementarity** | **Self 3' complementarity** |
| --- | --- | --- | --- | --- | --- | --- | --- | --- | --- |
| **Forward primer** | CCCCCTGGACAAAGACTGAC | Plus | 20 | 360 | 379 | 59.96 | 60.00 | 3.00 | 1.00 |
| **Reverse primer** | TCGTTTACGGCGTGGACTAC | Minus | 20 | 444 | 425 | 60.11 | 55.00 | 5.00 | 3.00 |
| **Product length** | 85 | | | | | | | | |

**Primer pair 4**

|  | **Sequence (5'->3')** | **Template strand** | **Length** | **Start** | **Stop** | **Tm** | **GC%** | **Self complementarity** | **Self 3' complementarity** |
| --- | --- | --- | --- | --- | --- | --- | --- | --- | --- |
| **Forward primer** | CTGCATTCGAAACTGGCAGG | Plus | 20 | 256 | 275 | 59.83 | 55.00 | 6.00 | 3.00 |
| **Reverse primer** | GTCAGTCTTTGTCCAGGGGG | Minus | 20 | 379 | 360 | 59.96 | 60.00 | 3.00 | 0.00 |
| **Product length** | 124 | | | | | | | | |

**Primer pair 5**

|  | **Sequence (5'->3')** | **Template strand** | **Length** | **Start** | **Stop** | **Tm** | **GC%** | **Self complementarity** | **Self 3' complementarity** |
| --- | --- | --- | --- | --- | --- | --- | --- | --- | --- |
| **Forward primer** | GGGAGGAAGGCGATGAAGTT | Plus | 20 | 70 | 89 | 59.75 | 55.00 | 3.00 | 3.00 |
| **Reverse primer** | GGAGTTAGCCGGTGCTTCTT | Minus | 20 | 139 | 120 | 60.04 | 55.00 | 4.00 | 0.00 |
| **Product length** | 70 | | | | | | | | |

**Primer pair 6**

|  | **Sequence (5'->3')** | **Template strand** | **Length** | **Start** | **Stop** | **Tm** | **GC%** | **Self complementarity** | **Self 3' complementarity** |
| --- | --- | --- | --- | --- | --- | --- | --- | --- | --- |
| **Forward primer** | CGGTAATACGGAGGGTGCAA | Plus | 20 | 153 | 172 | 59.82 | 55.00 | 5.00 | 2.00 |
| **Reverse primer** | AAGGGCACAACCTCCAAGTC | Minus | 20 | 468 | 449 | 60.18 | 55.00 | 3.00 | 1.00 |
| **Product length** | 316 | | | | | | | | |

**Primer pair 7**

|  | **Sequence (5'->3')** | **Template strand** | **Length** | **Start** | **Stop** | **Tm** | **GC%** | **Self complementarity** | **Self 3' complementarity** |
| --- | --- | --- | --- | --- | --- | --- | --- | --- | --- |
| **Forward primer** | AGTCGGATGTGAAATCCCCG | Plus | 20 | 221 | 240 | 59.82 | 55.00 | 7.00 | 2.00 |
| **Reverse primer** | TCAAGGGCACAACCTCCAAG | Minus | 20 | 470 | 451 | 60.18 | 55.00 | 3.00 | 1.00 |
| **Product length** | 250 | | | | | | | | |

**Primer pair 8**

|  | **Sequence (5'->3')** | **Template strand** | **Length** | **Start** | **Stop** | **Tm** | **GC%** | **Self complementarity** | **Self 3' complementarity** |
| --- | --- | --- | --- | --- | --- | --- | --- | --- | --- |
| **Forward primer** | TAATACGGAGGGTGCAAGCG | Plus | 20 | 156 | 175 | 60.18 | 55.00 | 4.00 | 2.00 |
| **Reverse primer** | CGGGGATTTCACATCCGACT | Minus | 20 | 240 | 221 | 59.82 | 55.00 | 7.00 | 1.00 |
| **Product length** | 85 | | | | | | | | |

**Primer pair 9**

|  | **Sequence (5'->3')** | **Template strand** | **Length** | **Start** | **Stop** | **Tm** | **GC%** | **Self complementarity** | **Self 3' complementarity** |
| --- | --- | --- | --- | --- | --- | --- | --- | --- | --- |
| **Forward primer** | CGAAACTGGCAGGCTAGAGT | Plus | 20 | 263 | 282 | 59.75 | 55.00 | 5.00 | 2.00 |
| **Reverse primer** | CTCAAGGGCACAACCTCCAA | Minus | 20 | 471 | 452 | 60.18 | 55.00 | 3.00 | 0.00 |
| **Product length** | 209 | | | | | | | | |

**Primer pair 10**

|  | **Sequence (5'->3')** | **Template strand** | **Length** | **Start** | **Stop** | **Tm** | **GC%** | **Self complementarity** | **Self 3' complementarity** |
| --- | --- | --- | --- | --- | --- | --- | --- | --- | --- |
| **Forward primer** | CGGTCTGTCAAGTCGGATGT | Plus | 20 | 211 | 230 | 59.76 | 55.00 | 3.00 | 1.00 |
| **Reverse primer** | TTTACGGCGTGGACTACCAG | Minus | 20 | 441 | 422 | 59.76 | 55.00 | 5.00 | 1.00 |
| **Product length** | 231 | | | | | | | | |
