# Supplementary material for: Biogenic gold nanoparticles conjugated with rhizobacteria enhance tomato growth and suppress pathogen infection
Source: Front Microbiol. 2026 May 5;17:1758150. doi: 10.3389/fmicb.2026.1758150 (PMC13183646; doi:10.3389/fmicb.2026.1758150)
Supplement: Supplementary file 4 [file Table_3.docx]

**Table S3.** Spearman’s rank correlation between log₁₀(Sr_CFU + 1) and log₁₀(Ep_qPCR) across three growing seasons using treatment mean values (n = 4).

| **Parameter** | **Season 1** | **Season 2** | **Season 3** |
| --- | --- | --- | --- |
| Spearman’s rank correlation (rs) | -0.2 | -0.4 | -0.4 |
| Coefficient of determination (r²) | 0.04 | 0.16 | 0.16 |
| P-value | 0.9845 | 0.6788 | 0.6788 |
| Covariance | -0.3333 | -0.6667 | -0.6667 |
| Sample size (n) | 4 | 4 | 4 |

Spearman’s rank correlation analysis was performed using the **mean values of four treatments (n = 4)** for each growing season to assess the relationship between *Stenotrophomonas rhizophila* rhizosphere populations [log₁₀(Sr_CFU + 1)] and *Erwinia persicina* abundance [log₁₀(Ep_qPCR)]. Negative rs and covariance values indicate an inverse relationship between rhizobacterial persistence and pathogen load. None of the correlations reached statistical significance (P > 0.05).
